# Tissue-Specific Expression and Regulatory Networks of Pig MicroRNAome

**DOI:** 10.1371/journal.pone.0089755

**Published:** 2014-04-03

**Authors:** Paolo Martini, Gabriele Sales, Mattia Brugiolo, Alessandro Gandaglia, Filippo Naso, Cristiano De Pittà, Michele Spina, Gino Gerosa, Francesco Chemello, Chiara Romualdi, Stefano Cagnin, Gerolamo Lanfranchi

**Affiliations:** 1 Department of Biology, University of Padova, Padova, Italy; 2 CRIBI Biotechnology Centre, University of Padova, Padova, Italy; 3 Department of Cardiac, Thoracic and Vascular Sciences, University of Padova, Padova, Italy; 4 Department of Biomedical Sciences, University of Padova, Padova, Italy; University of Turin, Italy

## Abstract

**Background:**

Despite the economic and medical importance of the pig, knowledge about its genome organization, gene expression regulation, and molecular mechanisms involved in physiological processes is far from that achieved for mouse and rat, the two most used model organisms in biomedical research. MicroRNAs (miRNAs) are a wide class of molecules that exert a recognized role in gene expression modulation, but only 280 miRNAs in pig have been characterized to date.

**Results:**

We applied a novel computational approach to predict species-specific and conserved miRNAs in the pig genome, which were then subjected to experimental validation. We experimentally identified candidate miRNAs sequences grouped in high-confidence (424) and medium-confidence (353) miRNAs according to RNA-seq results. A group of miRNAs was also validated by PCR experiments. We established the subtle variability in expression of isomiRs and miRNA-miRNA star couples supporting a biological function for these molecules. Finally, miRNA and mRNA expression profiles produced from the same sample of 20 different tissue of the animal were combined, using a correlation threshold to filter miRNA-target predictions, to identify tissue-specific regulatory networks.

**Conclusions:**

Our data represent a significant progress in the current understanding of miRNAome in pig. The identification of miRNAs, their target mRNAs, and the construction of regulatory circuits will provide new insights into the complex biological networks in several tissues of this important animal model.

## Background

Basic research and knowledge of human development, physiology, and pathology are closely tied by the use of suitable model organisms. Even if mouse and rat are the two mammals most used as human model organisms, many of their physiological parameters such as size, feeding, and respiratory rate are actually different from man. Furthermore, rodent genomes are evolving at a faster rate than the human genome [1]. The pig, despite a more expensive farming and a longer gestation period (114 days vs 20 days for the mouse), is a model organism that can overcome these problems thanks to several similarities with humans. In particular, the size of organs and various anatomical features, as well as physiology and organ development, are very similar in the two species allowing the use of the pig as model to study different important issues such for example pathologies affecting the cardiovascular [2], [3], gastrointestinal [4], and neuronal [5] systems, eyes [6] or muscles [7] or problems related with organ transplantation [8], [9]. Indeed, the pig has become the most important species for the production of xenografts to overcome the growing gap between request and availability of human organs suitable for transplantation [8], [10], [11]. Moreover, many varieties of pigs play a relevant role in the economy of human feeding, opening issues on food safety where it is used to study host-pathogen interaction [12].

Despite this well-documented importance, the knowledge about the genome organization, gene expression regulation, and molecular mechanisms underlying the physio-pathological processes of the pig are still far from the knowledge we have achieved for the mouse and rat. More than 90% of the porcine genome has been completely sequenced by the Swine Genome Sequencing Consortium [13]. Detailed information on the porcine genome, together with emerging transgenic technologies, will enhance our possibilities to create specific and useful pig models. Recently, the atlas of DNA methylomes in porcine adipose and muscle tissues was published [14] and a great effort was made to associate the genome sequence knowledge to studies faced at gene expression analysis. Many of these studies were focused on swine immune system [15]–[19], while a genome wide expression analysis in different tissues has been described [20]. In this scenario, the knowledge of miRNA expression, tissue specificity, and regulation acquire a fundamental role in the study of the transcriptome plasticity of the pig.

MicroRNAs are small non-coding RNAs that regulate gene expression in animals, plants, and protozoa. Transcribed from genomic DNA as long hairpins (pri-miRNAs), they are processed in the nucleus and exert their function in the cytoplasm where one strand of the ∼22 nucleotide duplexes is loaded in the RISC complex leading to mRNA translational repression and/or mRNA destabilization and degradation [21].

Recently, many studies have been directed to pig tissue-specific miRNA repertoires using deep-sequencing approaches. McDaneld [22] and Zhou [23] identified genes expressed in porcine skeletal muscle and predicted the miRNAs that may target these genes. Liu and colleagues discriminated between oxidative and glycolytic skeletal muscles identifying miRNAs that play essential roles in the phenotypic variations observed in different muscle fiber types [24]. Siengdee [25], McDaneld [26] and Huang [27] detected miRNA and mRNA involved in skeletal muscle development. MiRNA expressed in the kidney of different porcine breeds were analyzed, leading to the identification of breed-specific miRNAs, which could be potentially associated to specific phenotypes [28]. Moreover, miRNAs expressed by teeth have been studied as tools for investigating the molecular mechanism of tooth development [29] and those expressed by the pig intestine tracts [30] could be useful to study human pathologies of this organ due to the similarity among the two species. Also in the pig brain the miRNA repertoire was studied, leading to the identification of miRNAs that are specifically activated during brain development [31]. MiRNAs expressed during embryonic life in testis, ovary and spermatic cells of the pig were discussed in recent papers providing a valuable resource for investigators interested in the regulation of embryonic development in pigs and other animals [32]–[36]. Pituitary gland is important for homeostasis through specific hormone secretion: Hongyi and colleagues showed that cells of this gland produce a lot of miRNA involved in both development and physiology of this organ [37]. Li and colleagues demonstrated, in a comparison of muscle and adipose tissues of the pig, that a complex regulatory network may underlie the subcutaneous fat development due to the great diversity of miRNA composition and expression levels found in the two tissues [38].

To obtain better insight into the biological function of this class of small RNA molecules, the identification of all miRNAs expressed from the pig genome is essential to monitor their activity and function in different tissues and to fully identify their potential mRNA targets [39]–[42].

The study presented in this paper was aimed at contributing to a complete view of the pig miRNome and to improve knowledge of miRNA function in different pig tissues. First, we systematically analyzed the pig genome to identify putative pre-miRNA structures. In second instance, we experimentally identified true positive pre-miRNAs, i.e. those producing mature sequences, using modified RNA-primed Array-based Klenow Extension (RAKE) approach [43], [44] and RNAseq technologies with a pool of RNAs from 20 different tissues. This work is expanding the information on miRNA-tissue knowledge as it was reported up to now by Chen's [39] and Xie's [40] papers, who analyzed three or ten individual porcine tissues. The experimental identification of this large set of miRNAs and mRNAs allowed the concurrent characterization of the relationships between miRNA isoforms (isomiR) and between miRNA and miRNA star (miRNA*), and the analysis of tissue specific transcriptional regulatory networks.

The bioinformatic predictions of miRNA targets is a research field of a great expansion with a growing number of tools becoming available [45]. However, the rate of false positive results obtained with these tools is still very high, so we reasoned that the correlation between expression of miRNA and predicted targets, in their common physiological context, would be a useful strategy to support their connection and thus the functional relevance of predicted pairing. Both miRNA target prediction and mRNA/miRNA tissue expression provide novel or revised miRNAs description, substantially improving the view of miRNA-directed regulation in pig tissues.

## Results

### Bioinformatic prediction of pre-miRNAs

The identification of putative pre-miRNAs was made following two complementary approaches. The first approach was meant at identifying conserved miRNA through the comparison of the pig genome sequence with the collection of miRNAs identified in other species. The second approach was a *de novo* identification based on the prediction of the putative pre-miRNAs structures in the porcine genome.

**Figure 1:**
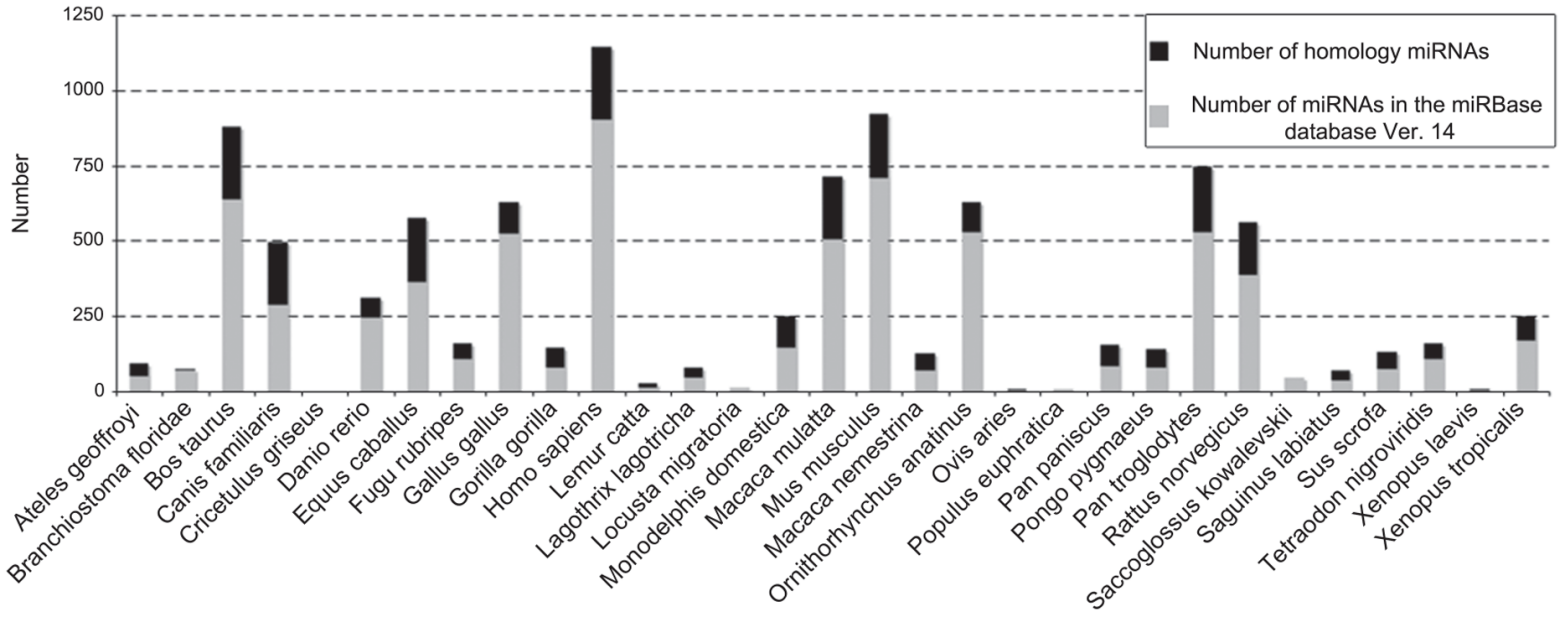
Number of conserved miRNAs. For each indicated species, the grey bars indicate the total number of miRNAs present in the miRBase db (version 14), whereas the black portion indicates the number of miRNAs with conserved seed region and at least 50% similarity of the pre-miRNA sequence with the sequence of the pig genome. Lower percentage of miRNAs with homology to pig genome are seen in invertebrates (e.g. *Saccoglossus kowalevskii* 2.22%; *Branchiostoma floridae* 2.74%), while higher percentages are found in primates.

We found that 39.8% of miRNAs in miRBase database (Ver. 14) are putatively conserved in the pig genome. We call conserved a genomic sequence that has a perfect match of the ‘seed region’ and at least 50% similarity to the pre-miRNA sequence. A small percentage of conserved miRNAs show similarities to invertebrate sequences (*Saccoglossus kowalevskii* 2.22%; *Branchiostoma floridae* 2.74%) while the great majority derive from primates (Figure 1). The percentage of pre-miRNAs putatively conserved in *Sus scrofa* was correlated with the phylogenetic distance between pig and other species. Even if there is an odd distribution of the number of discovered miRNA along the phylogenetic tree (Figure 1) still, it appears that, as the phylogenetic distance increases, the percentage of conserved miRNAs decreases (Pearson correlation: −0.53) (Table S1).

The *de-novo* identification of miRNAs was based on the recognition in the whole pig genome of hairpins that are compatible with the canonical pri-miRNA secondary structure. This analysis resulted in 10,618,726 distinct candidate pri-miRNAs that, filtered with the triplet SVM algorithm (probability score higher than 0.998), produced 10,239 probable pre-miRNA hairpins (Table A in Table S2) [46]. Finally, the sequence of Drosha binding site was identified through the Microprocessor SVM algorithm [47], obtaining 5,100 unique entries (SVM score <0, Table B in Table S2).

### Experimental identification of mature miRNA sequences

A series of microarrays, based on the RAKE approach, was used to experimentally identify mature miRNAs predicted by bioinformatic approaches (conserved and *de-novo* computationally identified pre-miRNA) [43], [44]. This experiments has two important consequences: first it leads to the identification of true positive pre-miRNA among all predictions and second it makes possible the identification of the mature miRNA sequences originated from pre-miRNAs. In fact, computational analyses are unable to predict the correct boundaries of the mature miRNAs. However, RAKE technology is able to identify only one of the ends of the mature sequences from each microarray experiment (see methods). This drawback was turned into an advantage because, to reconstruct a mature miRNA sequence, the independent experiments to determine 3′-end and 5′-end produce a statistically conserved signal compatible with the miRNA dimension.

Two microarrays (GPL13319 and GPL13320) with 90,000 probes each were synthesized to determine the 3′-end of miRNAs. Microarray probes were organized in tiling paths of 44 probes complementary to every predicted pre-miRNA. 22 probes, each shifted by one nucleotide, span each arm of the stem-loop structure of the predicted pre-miRNA (Protocol S1). These two platforms were used to screen 6,129 predicted pre-miRNAs (conserved and *de-novo* predictions) and the 215 known miRNA sequences collected from miRBase and the literature [26], [27], [48]–[50], using a pool of small RNAs prepared from 20 different tissues of the pig (Protocol S1). 3′-ends of the mature miRNAs were automatically annotated by processing raw microarray data through a custom algorithm (Peaks identification) (see Methods). We identified 1,875 responsive probes (i.e. 3′-ends) including cases of alternative 3′-ends on the same pre-miRNAs (Table C in Table S3).

**Figure 2:**
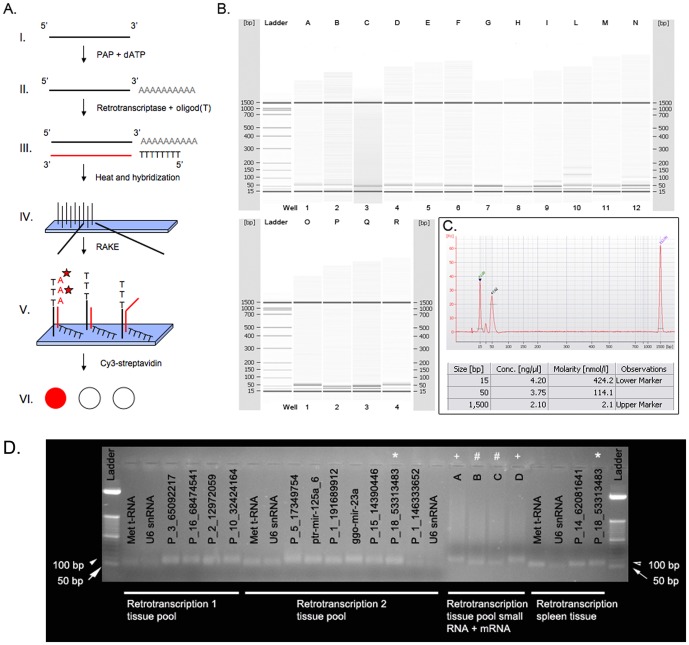
MicroRNA identification. A. Description of the method used to identify the 5′-end of the miRNAs. The small-RNAs (I) were subjected to a poly-adenylation step (II) and then to retrotranscription (III), using a 3′-degenerated oligod(T) primer to avoid long tails at the end of the cDNAs that could create problems in the hybridization step (V). We used a retrotranscriptase lacking terminal transferase activity to preserve the natural 5′-end of miRNAs. Produced cDNAs were probed to the microarray and used as primers in extension reactions performed with the Klenow polymerase and dATP-biotin (A-★) (V). Retrotranscribed miRNAs whose 3′-ends perfectly match a specific probe are extended (V). Only perfectly hybridized probes allow the incorporation of biotinylated dATP, producing a fluorescent signal due to the successive incubation of microarrays with Cy3-strepatvidin (VI). B. Gel-like images for the PCR amplified miRNAs. Samples I, L, and N showed multiple bands compatible with the dimensions of pre-miRNA, while samples A and D are bigger than expected (see Protocol S1). C. Electropherogram for the sample G. Peaks are evident for lower and upper markers, for the amplified miRNA (50 bp), and for the ladder (25 bp). The table indicates the concentration and size of the peaks identified in the electropherogram. D. Agarose gel electrophoresis of miRNA specific PCR products. Only primers A, B, C, D were used to amplify miRNA from samples containing mRNA while others are amplified from small-RNA purified population. MicroRNA amplification from mRNA containing samples is evidenced, but primer cross-hybridization with mRNA sequences causes the smear over the specific band. This pinpoints the necessity of miRNA purification for miRNA specific PCR amplification. Upper symbols (*, #, +) indicate the same amplicon and below the gel are indicated starting material used in the retrotranscription reaction. The letters and names over the gel lines correspond to the primers listed in the table SM2 of Protocol S1.

Pre-miRNA sequences, positive for the presence of a 3′-end, were used to design a new microarray platform (GPL13321) to detect miRNA 5′-end. The definition of the 5′-end represents a critical step because it defines miRNA seed region. This novel platform was composed by a tiling of 16 probes spanning the putative 5′-terminus of each 3′-end positive pre-miRNAs. Differently from 3′-end identification, the same 20-tissue RNA pool was first retrotranscribed and then hybridized to the described microarray (Figure 2 A). The Peaks identification algorithm was able to identify 2,120 responsive probes for the 5′-ends of miRNAs with 65% significance and of 240 with 58% significance, for a total of 2,360 responsive probes (Tables D and E in Table S3). Since the 5′-end identification is critical, we decided to reduce the significance threshold and to re-evaluate the results of the bioinformatics prediction in terms of miRNA expression measured by RAKE. The combination of bioinformatic prediction and expression studies with the tiling microarrays (3′- and 5′-end) resulted in the final identification of 1,459 candidate miRNA sequences corresponding to 1,102 hairpin sequences. Of these, 215 correspond to previously known miRNAs, 226 to conserved miRNAs (15.49%), and 1,018 represent novel potential miRNAs (69.78%) (Tables F and G in Table S4).

### Validation of RAKE results through RNA-seq experiments

To strengthen our results, we compared RAKE data with different RNA-seq datasets. We used five public RNA-seq datasets of various pig tissues (GSE28169, GSE14584, GSE17885, GSE24443, GSE30334 [33], [41], [51]) and three different RNA-seq experiments performed in our laboratories using the Ion Torrent technology with RNA from pig adipose, liver, heart, kidney and skeletal muscle tissues. A total of ∼400 milion of reads, not matching with coding transcripts or other RNAs but miRNAs, were mapped on pre-miRNA sequences.

RAKE responsive hairpins were classified as high, medium and low confidence miRNA precursors according to sequence coverage by RNA-seq data. Of the 1,102 predicted hairpins initially confirmed after RAKE, 299 were classified as high confidence pre-miRNAs resulting > = 10 the coverage of their mature sequence, while 254 hairpins were classified as medium confidence pre-miRNAs with an averaged coverage of the mature sequence > = 3 and <10. 424 miRNAs originate from high confidence and 353 from medium confidence pre-miRNAs (Tables H and I in Table S4). Hairpins identified as responsive for the presence of mature miRNA only by RAKE have been classified as low confidence. Comparing RAKE result with those obtained by Chen and colleagues [39] and Xie and colleagues [40] we obtained an over imposition of 42% and 49% respectively (Tables J and K in Table S5). This over imposition increases if it is considered miRNA they detected basing on miRBase information (75% and 56% respectively) indicating the importance of stringent criteria in miRNA identification process. Only high confidence miRNA sequences were considered for further analyses to describe gene regulative network in different pig tissues.

### PCR-based validation of RAKE results

After the RNA-seq experiments we ended up with three classes of miRNA defined as high, medium and low confidence. To check experimentally the reliability of this classification, we tested 30 RAKE-positive miRNA candidates by PCR-based amplification (Protocol S1). 23 were confirmed by the amplification of a single specific product with the expected dimension, and this number increases to 26 if we include amplifications compatible with pre-miRNA products and no aspecific amplicons (Figure 2 B, 2 C, 2 D and Protocol S1). PCR amplification from non-enreached miRNA (material containing mRNA) does not allow the production of a single band, probably because of the cross-hybridization of primers with mRNA sequences (Figure 2 D). Of the 23 positive miRNAs, 10 belong to the high confidence set, 2 to the medium and 11 to the low confidence set demonstrating that also medium and low confidence sets contain real miRNA (Protocol S1). Moreover, qRT-PCR experiments demonstrated the reliability of RAKE for measuring miRNA expression levels (see next section).

### Tissue-specific microRNA expression signatures

**Figure 3:**
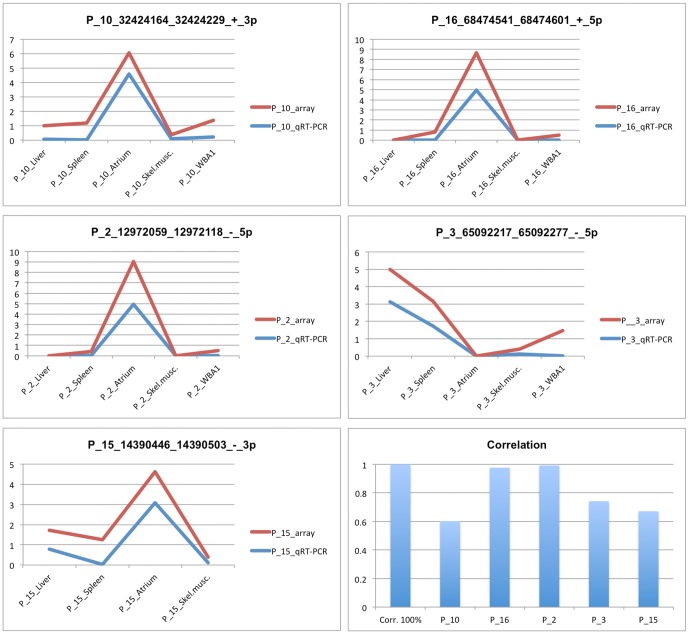
MicroRNA expression comparison. MicroRNA expression profiles from qRT-PCR (blue line) and microarray analyses (red line). The Y-axis represents miRNA quantity related to the average quantity in the considered tissues. Correlation between qRT-PCR and microarray expression profiles range from 0.6 to 0.99.

Before performing a genome-wide analysis, miRNA expression was validated by qRT-PCR. We considered five different miRNAs. Since the pig is an important animal model for pre-clinical studies, especially for cardiovascular diseases [52], four miRNAs highly expressed in the heart and one presenting a pronounced expression in the liver were chosen. Their expression was evaluated by qRT-PCR in five different tissues: liver, spleen, atrium, skeletal muscle, and white blood cells (Figure 3). We obtained a range of correlation between microarray and qRT-PCR results comprised between 0.6 and 0.99 (Figure 3). According to RNA-seq results, only one tested miRNA did not fall in the high confident class (P_16_68474541_68474601), nevertheless it showed a correlation between qRT-PCR and microarray results higher than 0.9. Bringing forward results about the integration of miRNA and mRNA expression it is interesting to notice that putative target genes of the four miRNAs highly expressed in the heart are enriched in the ubiquitin protein ligase binding capacity. This result is interesting since inhibition of the cardiac proteasome, here evidenced as potentially regulated through miRNAs, has been shown to be cardio protective under some circumstances [53].

**Figure 4:**
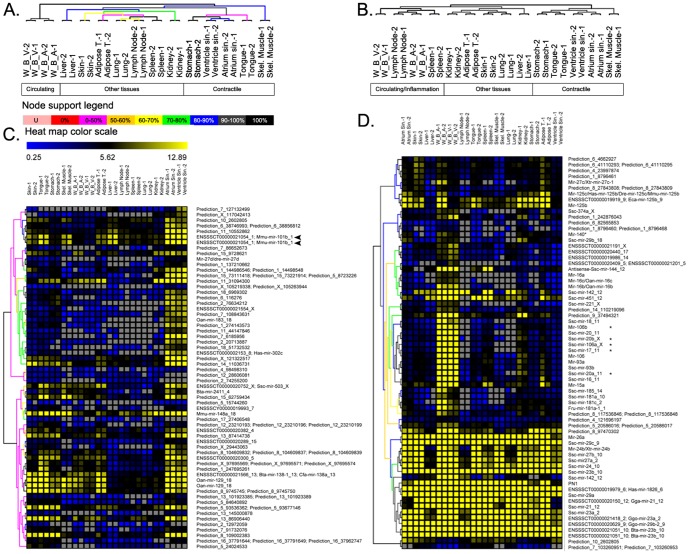
Cluster analysis of miRNA and mRNA expression signatures in 14 pig tissues. A. Clustering tree of pig tissue samples according to miRNA expression data. The color of the arms describes the statistical support for the nodes, based on data resampling, as quantified in the node support legend. Each experiment was performed in duplicate, with replicas indicated with numbers 1 and 2 in the sample name. B. Clustering tree of pig tissue samples according to mRNA expression data. C. Heat map of miRNA specifically expressed in heart (atrium and ventricle). Arrows indicate most up-regulated miRNA. D. Heat map of the miRNAs specifically expressed in white blood cells. * indicates components of mir-17 family. Grey squares indicate expression below the limit of detection. L.A. = Left Atrium; W.B.A. = White Blood Cells from Arterial blood; W.B.V. = White Blood Cells from Venous blood; L.N. = Lymph Node; S.M. = Skeletal Muscle; A.T. = Adipose Tissue; L.V.: Left Ventricle.

Next, we analyzed the expression pattern of the discovered miRNAs across 14 different tissues of the pig. Given the results of PCR validations, the microarray platform to profile miRNA expression was designed to cover all the miRNA types (high, medium and low confidence). MiRNA expression was evaluated using RAKE coupled with a spike-in based quantization method [54], [55] (Protocol S1) (GSE28140). This genome-wide study allowed the separation of tissues according to miRNA signatures. They cluster according to the structure, anatomical location, and physiological functions of tested organs, suggesting that the function of a miRNA could be inferred by the biology of tissue in which it is uniquely or mainly expressed. MicroRNA expression profiles divide pig tissues in three large clusters (Figure 4 A): a) tissues with contractile properties like heart atrium and ventricle, stomach, tongue, and skeletal muscle, b) circulating cells (white blood cells), and c) all other examined tissues (liver, skin, adipose tissue, lung, lymph node, spleen, and kidney). Gene expression pattern of the stomach is linked to contractile tissues, showing that the smooth muscle component is predominant over the epithelial tissue of the stomach wall, while the skeletal muscle shows an expression pattern more comparable to that of the tongue. The profile of white blood cell samples formed an out-group in the clustering tree, reflecting the specific features of these cells. The third cluster is composed of a group of tissues, such as lymph node, spleen, and adipose tissue. The similarity between spleen and lymph node samples reflects their important role for the immune system. MiRNA analysis of human normal tissues showed that these tissues have a very similar expression profiles also in man [56]. Hierarchical clustering of mRNA profiles (Protocol S1) (GSE27853) shows a tissue stratification that closely resembles those obtained for miRNAs (Figure 4B), with tissues involved in immunity that group together.

Many miRNAs are expressed across several tissues, but large sets of miRNAs were found to have, instead, a tissue-specific expression. Five different clusters of miRNAs appear to be restricted to white blood cells, myocardium, skeletal muscle, adipose tissue and liver respectively. Hereafter we discuss the miRNA clusters specific for white blood cells (WBC) and myocardium because of the importance of these two tissues in cardiocirculatory system [57].

#### Myocardium

Despite structural and functional similarities with human myocardium and the wide use of pig heart valves in cardiovascular surgery [58], [59], there are few genome-wide experimental identifications of miRNAs in the pig heart [60]. Our analysis identified a cluster of miRNAs preferentially expressed in atrium and ventricle with some overlap with those expressed in the stomach (Figure 4 C). Many components of this cluster are pig-specific miRNAs, and the most up-regulated is mir-101b, which appears to have an important role in heart function because it was found under-expressed in the ischemic reperfused myocardium in the rat model [61].

#### White Blood Cells

We found that miRNAs with higher expression in WBCs includes different miRNA families: mir-15, mir-17, mir-181, mir-23, mir-27 and mir-29 families. The mir-17 family is the one most enriched (p = 3.24 E-4; Table S6) and comprises mir-17, mir-18a, mir-19a, mir-20a, mir-19b-1 and mir-92-1. This family is expressed as polycistronic units, revealing a common regulatory mechanism [62], that is confirmed by the similarity of their expression profiles (Figure 4 D). Another gene family that we evidenced prevalently expressed in the WBCs is mir-29 (mir-29a, mir-29b, mir-29c; p = 2.19 E-4). This family is prevalently involved in different lymphomas [63] and its downregulation is associated with disease progression and poor prognosis of these tumors [64].

**Figure 5:**
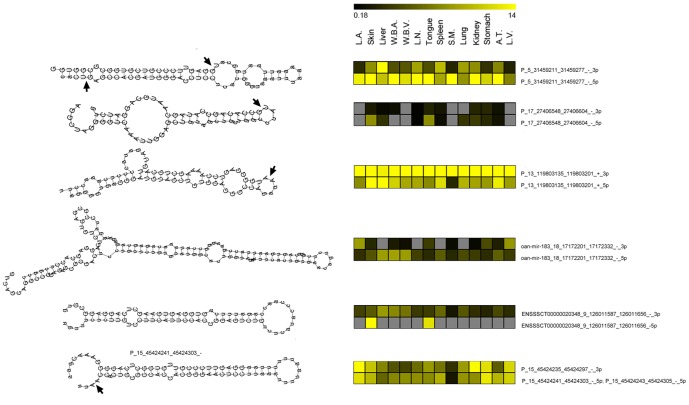
Structural features of pig pre-miRNAs and alternative expression profiles of derived miRNA-miRNA* pairs. The left column reports the pre-miRNA hairpin structures as defined by the RNAfold algorithm. For each pre-miRNA, uppercase letters indicate miRNA mature sequences and black arrows their alternative 5′- and 3′-ends. The right column shows the expression signatures of miRNA-miRNA* pairs derived from pre-miRNA hairpin depicted in the left column. Expression was monitored by microarray RAKE on 14 different pig tissues (for tissue symbols see description in the caption of figure 4). The color intensity scale reported on top of the heat maps is proportional to the concentration of miRNAs and varies from black (low concentration) to bright yellow (14 pM concentrated). Gray squares indicate concentration under detection limit. MicroRNA identifications are composed of features (letters and numbers) divided by underscores as explained below. The first feature is the miRNA name and could be P (standing for predicted and referring to the *de-novo* identification), the Ensembl, or the Ver. 14 miRBase identifications (homology identification). The second feature indicates the chromosome number where the pre-miRNA is located. The third and fourth features indicate the start and stop nucleotide positions of pre-miRNAs in the pig genome. The fifth feature (+ or −) shows the template DNA strand. The sixth feature (3p or 5p) distinguishes the two arms of pre-miRNA hairpins. MicroRNA and miRNA* show an opposite expression profile across the different tissues that support a different functional role for miRNA and miRNA* in specific tissues. For example, mir-183_18_17172201_17172332_-_3p is highly expressed in L.V and L.A., but not in the Liver, L.N., Spleen and Lung where its 5p counterpart is instead more expressed.

Interestingly, miRNA/miRNA* couples show a peculiar and different expression pattern across different tissues (Figure 5), indicating that the two members of the couples may have different functions and could be retained in the RISC complex according to the specific function exerted in different cellular environments.

Tissue-specific expression studies of microRNA provide clues for the regulatory mechanisms that regulate their expression but the comprehension of their function is facilitated by the discovery and association with their correct mRNA targets. To this aim, a new microarray platform was developed to obtain the profiles of all large RNA transcripts in pig tissues to be integrated with miRNA profiles.

### Integration of miRNA and mRNA expression signatures and identification of miRNA targets

We performed mRNA expression profiling on the same tissues selected for miRNA profiling. Expression profiles were obtained with a new oligonucleotide microarray platform (GPL13259) that covers the full repertoire of long RNAs transcribed by the pig genome (ENSEMBL ver. 64 and UniGene ver. 38). On the basis of sequence similarity, UniGene features that overlapped more than 40% an Ensembl transcript were discarded. After this filter, we obtained 40,267 UniGene clusters and 19,603 Ensembl transcripts (protein coding+pseudogenes+retrotransposed elements). Before proceeding with definitive microarray, different probes were computationally designed and the two best probes in terms of specificity and proximity to the 3′-end of the transcript were experimentally tested in a trial hybridization with a pool of RNAs prepared from 20 pig tissues (GSE28636). For each transcript with a duplicated probe, we selected the probe that was more responsive and specific on the basis of intensity of fluorescence in the hybridization test, as suggested by Kronick [65]. The definitive pig whole-genome microarray here used for gene expression analysis is composed of i) 17,048 replicated probes and 963 unique probes specific for Ensembl transcripts, ii) 11,363 replicated probes specific for UniGene clusters of length comprised between 778 and 1,348 nt, and iii) 28,790 unique probes specific for the remaining UniGene clusters. As a result, 98.2% of the probes of the Ensembl transcripts mapped within 2,000 nt from their 3′-end while the percentage for UniGene clusters reached 99.9%. With this design procedure, we were not able to produce specific probes for 114 UniGene clusters and 1,592 Ensembl transcripts. To overcome the still poor gene annotation of the pig genome, we tried to increase the number of annotated features on the microarray by mining description and protein annotations to associate to our probes. Basically, for those whom HUGO symbol was not present we mined the description available from Unigene database and retrieved (if present) additional gene or protein IDs. All IDs were manually curated (GPL13259).

The unsupervised hierarchical cluster analysis of mRNA expression profiles (Protocol S1) (GSE27853) showed a tissue stratification that closely resembles that obtained from miRNA expression (Figure 4 B), indicating that the two RNA population work synergistically in establishing tissue specificity.

We next integrated the mRNA and the miRNA profiles by matching the datasets obtained for each pig tissue. A major limitation in the network analysis of genetic circuits is the unavailability of mRNA and miRNA profiles from the same sample. The present study overcomes this limitation and provides fully compatible datasets for developing new algorithms, other than those published [66], to detect modulation of target mRNA expression by miRNAs. Using bioinformatic approaches based on the identification of the seed region matching the 3′-UTR of the mRNAs (TargetScan algorithm [67]), we associated the collection of pig miRNAs with their potential mRNA targets. Furthermore, the expression of the mRNAs was integrated with those of miRNAs to gain insight on the function of the identified miRNAs. The global interaction network was composed by 5,226 nodes (miRNAs and mRNAs) and 15,235 edges (interaction between miRNA and mRNA and between different mRNA; see Methods) (Table S7). Our objective was to identify miRNA and mRNA expression patterns that may contribute to maintain tissue functionality and specificity. We were able to build tissue-specific mRNA/miRNA interactive networks for each of the 14 porcine tissues by the anti-correlation of mRNA/miRNA expression patterns [66]. Moreover, the same approach was applied to identify long intergenic non-coding RNA (lincRNA) that can function as sponges for miRNA [68], [69]. We detected five different lincRNA that have and expression profile that highly correlate with different miRNA (Table S8).

### Tissue-specific regulatory circuits

**Figure 6:**
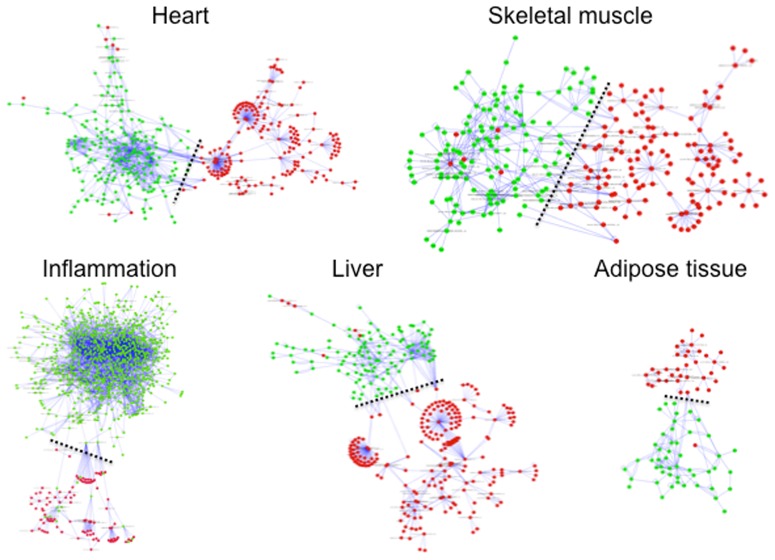
Tissue specific networks. All networks evidence a bipartite structure and dash line indicates bipartition point. In green is indicated the “permissive” part of the network connecting downregulated miRNAs and upregulated mRNA targets and in red the “repressive” part connecting upregulated miRNAs and downregulated target.

The integration of the tissue specific mRNA/mRNA and mRNA/miRNA interaction maps results in networks with a bipartite structure (Figure 6). One part of the network connects downregulated miRNAs and upregulated mRNA targets and we define it as “permissive” while the other part connects upregulated miRNAs and downregulated target mRNAs and we name it “repressive”.

**Figure 7:**
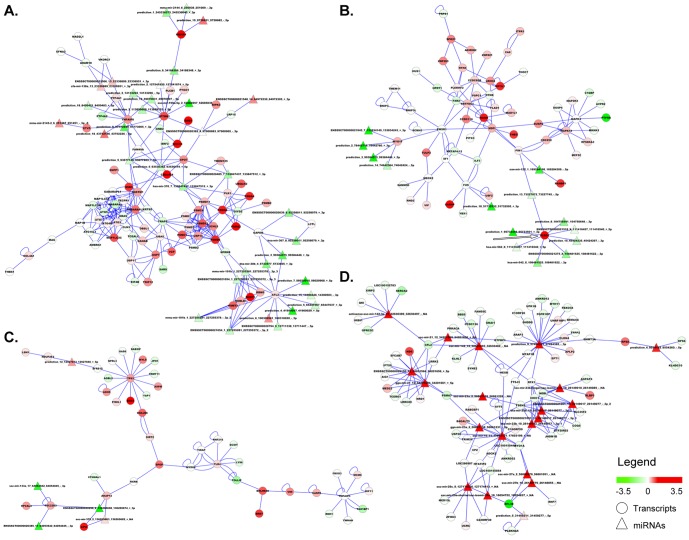
Modules of network describing tissues involved in inflammatory processes. A. Module 1 describes cell homeostasis through autophagy process. B. Module 2 can be described as the transcription regulation in inflammatory cells. An interesting gene of this module is PLBD1 (arrow) that codify for a phospholipase B precursor purified from normal granulocytes. The expression of this gene appears to be modulated in the module by 8 different miRNAs whose expression level is anti-correlated with that of the gene. C. Module 3 that contains different inflammatory pathways like those related to Toll-like receptor, NF-kB signaling and apoptosis. D. Repressive part of the general network is characterized by phosphor- and lipid biosynthetic processes and cytoskeleton/cell shape organization. Color coded are represented as log_2_ (sample/average of all samples). Circles are for mRNA while triangles for miRNAs. Edges indicate a relation between mRNA recovered from BioGRID database or between mRNA and miRNA according to TargetScan based target prediction.

The most complex network we identified was that related to tissues involved in inflammatory responses. It can be deconstructed in at least 18 functional modules. Here we will discuss modules containing at least 4 miRNAs since we are interested in mRNA regulation miRNA-dependent. Module 1 is made by 116 nodes and 276 edges (Figure 7 A) and, according to gene ontology analysis, it describes cell homeostasis through autophagy process. The subject of the second permissive module (Figure 7 B), (73 nodes and 110 edges) can be described as the transcription regulation in inflammatory cells. An interesting gene of this module is PLBD1 that codify for a phospholipase B precursor purified from normal granulocytes [70]. The expression of this gene appears to be connected in the module with 8 different miRNAs whose expression level is anti-correlated. The third module (48 nodes and 68 edges) contains different inflammatory pathways like those related to Toll-like receptor, NF-kB signaling and apoptosis (Figure 7 C).

The repressive part of the network involved in inflammatory responses (Figure 7 D) is characterized by phosphor- and lipid biosynthetic processes and cytoskeleton/cell shape organization.

We generally noted that miRNAs highly expressed in well-differentiated tissues, where the protein turnover is relatively slow, control processes associated with regeneration and proliferation. For example, positive regulation of apoptosis and developmental pathways are biological functions enriched (enrichment score 2.64 and 1.76 respectively) by mRNAs that are anti-correlated with miRNAs highly expressed in skeletal muscle (Table M in Table S9). Other significantly enriched categories are those describing the positive regulation of T cell activation, regulation of cell cycle and striated muscle cell differentiation (enrichment score are 1.34, 1.30 and 1.29 respectively) (Table M in Table S9). Another example of this relationship is given by the network related to heart. Regeneration ability is not maintained for this organ in the adult life [71], and in fact our results show that miRNAs highly expressed in heart are those related to (and thus inhibiting) ubiquitin-dependent catabolic processes and proteolysis (enrichment score 4.01) (Table N in Table S9). Conversely, up-regulated transcripts of the heart connected to downregulated miRNAs in the permissive network are involved prevalently in the maintenance of protein folding, cell vitality and mitochondrion respiration (Chaperon function, enrichment score 8.62; regulation of cell death, enrichment score 8.12; organelle envelope, enrichment score 8.01; Table O in Table S9).

In skeletal muscle, the permissive network is enriched with transcripts coding for proteins involved in muscle contraction and in sarcomeric Z-disc structure (Table P in Table S9) and shows a high anti-correlation with a group of miRNAs. Moreover, some important genes of the heart, such as the Ras-related associated with diabetes (*RRAD*), which may play an important role in cardiac antiarrhythmia [72], the ubiquinol-cytochrome c reductase core protein I (*UQCRC1*), which is involved in cardiomyopathies, and the solute carrier family 25 (mitochondrial carrier; phosphate carrier), member 3 (SLC25A3), whose deficiency is associated with hypertrophic cardiomyopathy [73] are inversely correlated with the expression of different miRNAs. The comparison of permissive networks for skeletal muscle and heart evidences that the heart specific network is characterized by genes involved in the formation of adherent junctions.

In conclusion, trough the integration of the interaction maps from the BioGRID database [74] and transcripts inferred as miRNA targets we produced networks that summarize the molecular interactions controlling the steady states of pig tissue in normal conditions.

## Discussion

### MicroRNA identification

According to the guidelines for miRNA annotation [75], a putative miRNA sequence should be supported by data demonstrating its evolutionary conservation and by evidence of expression in order to be recognized as a genuine miRNA. The collection of miRNAs presented here is supported by stringent bioinformatic criteria. Interestingly, the number of conserved miRNAs is related to the phylogenic distance between *S. scrofa* and the species considered in the evolutionary comparison (Figure 1). This indicates that miRNA functional role is maintained across phylogenetically close species, while species-specific miRNAs with their peculiar function may be lost during evolution.

We also adopted stringent criteria to experimentally validate the predicted miRNAs. RAKE and deep sequencing experiments allowed the identification of 299 hairpins (high confidence) that maturate functional miRNAs. Moreover, PCR-based validation of RAKE results was used to discuss the possibility to obtain mature miRNA from other pre-miRNA. Interestingly, with this approach we showed that different pre-miRNAs, in addition to those defined as highly confident, allow miRNA maturation. This demonstrates the stringency of the criteria adopted to produce our regulatory networks, where only fully validated miRNAs were considered and the interactions did not needed to be weighted down for false positive miRNAs.

### MicroRNA gene expression

MiRNA microarrays allow for massive parallel and relative measurement of all known miRNAs, but they cannot be used for absolute quantification. In fact, the short length of miRNAs makes difficult the proper selection of complementary probes that result in a high dynamic range of melting temperatures. Here we developed a new method that integrates the hybridization of miRNAs to a specific microarray with an enzymatic elongation reaction that can take place only after the establishment of a perfect match between miRNA and probe. Moreover, we introduced oligonucleotide spikes in the hybridization and enzymatic reaction, allowing the relative quantification of miRNAs and avoiding biases related to sequence, labeling, or hybridization. An alternative method for the absolute quantization of miRNAs was recently proposed [76]. Our method allows the detection of a comparable concentration of miRNA (10^−18^ moles to 10^−14^ moles in a linear range, see Protocol S1) but, at the same time, allows the control of quality and reproducibility of hybridization with the interpolation of the spike-in dependent curve. The performance of our method is independent from sequence differences because the labeling reaction that generates the signal for the positive hybridization of a miRNA molecule to a perfectly matching probe is due to the incorporation, by the Klenow enzyme, of labeled adenosines (biotin-dATP) and not to an enzymatic process performed before the hybridization.

The method here developed was subjected to qRT-PCR control by testing the expression of five miRNAs (Figure 3). The correlation between qRT-PCR and microarray expression was good demonstrating that profiles across different tissues through our microarray technique, based on a titration curve quantization, is reliable in measuring different miRNA expression across samples. Moreover, qRT-PCR analysis anticipates results of network analysis. In fact, four highly expressed heart-specific miRNAs were chosen for these validations. The association of putative mRNA targets to these heart miRNAs shows that they are enriched for transcripts codifying ubiquitin protein ligases, enzymes that are active in protein catabolism mediated by the proteasome. The inhibition of cardiac proteasome is cardioprotective [53], [77] and, as stated by May and colleagues [77], proteasome inhibition, and also the miRNAs involved in this process that we evidenced, could provide new therapeutic strategies to prevent cardiac fibrosis and progression of heart failure.

The RAKE method allowed us to analyze the expression signatures of 14 different pig tissues. They cluster according to anatomical proximity and functional similarity. Moreover, miRNA clusters are preferentially expressed in a tissue-specific manner suggesting a role for these miRNAs in the maintenance of specific molecular processes in the considered tissue (Figure 4). Some of the identified clusters are discussed below.

#### White Blood Cells

Recently, pig and human pathologies have been linked by the discovery that the H1N1 strain of influenza virus is able to infect humans with potential pandemic effects [78]. Therefore, it would be important to understand the immune system of these mammals and the molecular mechanisms that lead to viral infection. Moreover, the identification of miRNAs prevalently expressed in WBCs could be useful in the identification of immunological mechanisms that are important for the butchering industries. The mir-17 family showed the most pronounced expression in WBC (Figure 4D and Table S6). The over-expression of mir-17, 18, 19a, and 20 was demonstrated in tumors of the lung [79] and a second study reported the up-regulation of the miR-17-92 cluster in B-cell lymphomas [62]. We found, on the contrary, that mir-17, 18, and 20a are not expressed in normal lung (Figure 4 D). Taken together, these data support the importance of these miRNAs for the function of the immune system. The study of these miRNAs could be a good basis for the identification and analysis of potential immuno-modulatory effectors in immuno-mediated diseases like multiple sclerosis, where a down-regulation of miR-17 and miR-20a associated with T-cell activation was demonstrated [80], or like inflammatory myopathies. A possible important advancement in this topic will be the identification of interactions between the miRNAs of these paralogue families and their targets.

#### Contractile Tissues

The proximity between myocardial and smooth muscles can be explained by their common properties. Smooth muscle forms the major contractile elements of the viscera, especially those of the respiratory and digestive tracts, the blood vessels, and the stomach wall. Cardiac muscle has many properties in common with smooth muscle. For example, it is innervated by the autonomous system and contract spontaneously. Presumably, cardiac muscle evolved as a specialized type from the general smooth muscle of the circulatory vessels [81]. This could explain the similarity of miRNA profiles of atrium and ventricle. The presence of the tongue-specific miRNAs in the same contractile cluster (Figure 4 A) can be explained by the fact that this organ is mainly composed of striated muscles (16 different).

#### MicroRNA/MicroRNA*

miRNA biogenesis proceeds via an obligate step that produces small RNA duplexes, and the small RNAs (miRNA and miRNA*) are necessarily produced initially by transcription at a 1∶1 ratio. However, the final ratio of mature miRNA and miRNA* is asymmetric, sometimes with an altered proportion up to >20∶1 [82]. Although one member of the miRNA/miRNA* pair is usually dominant, pre-miRNA hairpins are nevertheless evolutionarily selected to produce a specific amount of functional miRNA* species [83]. The lack of correlation between the expression rates of paired miRNA and miRNA* demonstrates that, although deriving from the same pre-miRNA, they are present in the cell at different concentrations, probably reflecting different biological functions. A simpler alternative interpretation is that certain miRNA* strands are degraded more slowly than others. Nevertheless, the alternate expression profile that we measured for miRNA/miRNA* pairs in different pig tissues (Figure 5) supports the idea of a specific biological function for each member of the couple, guided by their tissue specific expression and a completely different seed sequence.

### Prediction of miRNA targets

For a better understanding of miRNA–target interaction there is a need for studies encompassing both mRNA and miRNA expression measured in the same context. Here we integrated mRNA and miRNA signatures obtained from the same tissue sample, establishing tissue-specific circuits and discussing their possible integration and regulation. This analysis was focused on the specific mechanism of miRNA action based on mRNA degradation. We integrated mRNA gene expression, gene/gene and miRNA target interactions in comprehensive networks, evidencing circuits involved in the functional maintenance of different tissues. A new microarray platform was developed, containing the best probes that are able to detect the whole pig transcriptome.

Recently, a new microarray platform was published based on 52,355 expressed sequences comprising miRNAs in miRBase ver. 15 of pig, cow, human and mouse [20]. Differently from this platform that was constructed spanning 22 probes along the whole length of the transcripts, our platform is build instead with probes designed in the 3′-UTR of each transcript. We think that our platform is more suitable for expression studies in the pig. It is well established in fact that the 3′-UTR are specific traits of each transcript and therefore ideal to distinguish mRNA isoforms. Furthermore, miRNA activity is prevalently based on the interaction with 3′-UTR region of target mRNAs [84].

A growing number of studies have assigned to miRNAs a fundamental role in the regulation of a variety of cell processes, and many of those identified in this study are well-positioned to regulate gene expression in different pig tissues as they presented a negative correlation with the expression of their predicted targets. Several observations showed that miRNAs are essential for the normal development of mammals. Here, we suggest that they are also important in the maintenance of normal tissue function. For example, we found that genes involved positive regulation of apoptosis and developmental pathways are targeted by miRNAs in the skeletal muscle (Table M in Table S9). We reason that in adult skeletal muscle there is no need for constant expression of important transcription factors and proteins involved in chromatin regulation that are expressed during muscle development. In the heart, where the regenerative process is limited because heart muscle cells are terminally differentiated, the importance of cell structure maintenance is fundamental. From our analyses it appears that heart muscle cells control the protein degradation pathway trough ubiquitinization by upregulating miRNAs targeting mRNAs for ubiquitinating enzymes and activating chaperons to control protein folding (Table N in Table S9). Inhibition of the cardiac proteasome has been shown to be cardioprotective under some circumstances, indicating the clinical potential for understanding its function [53]. Among miRNAs preferentially expressed in the heart (Figure 4) mir-148a, mir-101, and mir-138 are particularly important. Mir-101, which we found up-regulated in normal heart, was found instead down-regulated in patients with atrial fibrillation [85]. Furthermore, we showed that different genes involved in cardiomyopathies like emerin (*EMD*), nuclear factor of kappa light polypeptide gene enhancer in B-cells 1 (*NFKB1*) are under the control of specific miRNAs.

Even if skeletal muscle and heart are both striated muscles they differ in the architecture. The skeletal muscle functional unit is a cellular syncytium where different myogenic cells are fused to form a multinucleated myofibril while heart tissue is composed by different branched cells. Different heart cells communicate and coordinate their action through junctions that allow the electrical network. This structural difference was also evidenced in the specificity of gene expression. Heart permissive network was characterized by the expression of genes transcribing for elements of cell-junction and adherens junction that are not expressed in the skeletal muscle (Table O in Table S9).

Correlation analysis pointed the importance of different lincRNA in miRNA regulation. Interestingly, one miRNA cluster (composed by miR-192 and miR-194a) appears to be regulated by the same lincRNA (lincRNA 555, ENSSSCT00000033052). Moreover, miR-92a, one component of the miR-17-92 cluster important for the function of the immune system, could be regulated by lincRNA 841 (ENSSSCT00000035481, Table S8). The fact that lincRNA 841 is more expressed in white blood cells than in other tissues could sustain its functional importance in this tissue.

## Conclusions

How the differential expression of large numbers of molecular effectors such as mRNAs and miRNAs influence tissue function remains a challenging issue for systems biology. To approach properly this problem in the pig model, we decided first to increase the knowledge and characterization of the pig miRNAome. We have used stringent parameters for the prediction of phylogenetically conserved and *de-novo* identified miRNAs, providing strong evidence for 1,102 hairpins. Among these, we considered 299 hairpins as high confident, while 254 as medium confident. Moreover, positive results of directed PCR tests for putative miRNA sequences not included in these two groups indicate that more RAKE candidates can be confirmed as functional pre-miRNA in analyzed tissues, improving the knowledge of miRNAs in the pig as well as in other mammals.

The quantification of miRNA expression in different normal tissues is a first important step to investigate their associated functions. Moreover, it can provide an essential baseline to analyze the variation of miRNA abundance in pathological conditions. Our study supports previous evidence [67] that a single miRNA can bind to and regulate many different mRNA targets and, conversely, that several different miRNAs can interact with and cooperatively modulate a single mRNA target (Figure 7). The identification of miRNAs, their target mRNAs, and the construction of regulatory circuits will provide new insights into complex biological procedures of this important animal model, evidencing that complex regulatory networks mediate many facets of eukaryotic cell function in a tissue-specific way (Figure 6).

## Methods

### Ethics statements

This study was carried out in strict accordance with the recommendations in the Guide for the Care and Use of Laboratory Animals of the Italian Ministery of Health. Animals were provided by CISRA Institute (120TO025—ASL 3 Collegno, Torino, Italy). Pig used were 12 months old with weights of 59.7±2.3 kg. All animals were screened for Swine Vesicular Disease and vaccinated for Aujezky, Parvovirosis and Swine Erysipelas. The University of Padova was authorized to use pigs for experimental purposes (art. N. 12 D.Lgs.116 27.01.1992) under project registration number 27/08 C16, authorized by Italian Ministery of Health, according to the principles given by ISO 10993-2. All efforts were made to minimize animal suffering. Animals were sedated with midazolam (0.3 mg/kg), medetomidine (15 mg/kg), and ketamine (10 mg/kg), administered into the neck muscles. Anesthesia was induced with isoflurane in oxygen and maintained via a circular breathing system. After the injection of alfentanil (1–1.5 mg/kg/min) to maintain general anesthesia, euthanasia was performed changing inhalation gas from oxygen to carbon dioxide.

### RNA sample extraction

RNA samples (total RNA and small RNAs) were extracted from analyzed tissues of three different, not inbred pigs and kept at −80°C until use. Before performing all experiments, the three samples from the same tissue were pooled and miRNA selected through flashPAGE instrument (Protocol S1).

### Bioinformatics approaches for miRNA gene prediction and RAKE peaks identification

#### Conserved miRNA

Homologous conserved miRNAs were searched for in the pig genome (*S. scrofa*, assembly 9, April 2009) by the Blast algorithm [86], filtering out miRNAs in the miRBase database presenting similarity lower than 50% of their pre-miRNA sequence and no perfect match of the seed region.

#### De-Novo miRNA Identification

The first step of the *de-novo* bioinformatic prediction of miRNA genes consisted in the search for all the locally stable RNA secondary structures. To this end, we ran the RNALfold software [87] over the genome sequences of *Sus scrofa*. The two DNA strands were processed independently. We filtered the results by selecting structures longer than 54 and shorter than 217 bases, with a free energy lower than −27 kcal/mol and presenting at most 2 loops. A loop was defined as an unpaired region with a length between 3 and 30 nt.

In the second step, we followed the approach described by Xue [46], based on a support vector machine (SVM) to identify specific sequence and structure features connected to genuine miRNAs. To train the model, we selected as positive training set, the known miRNA sequences collected in the miRBase (Ver. 14) for the following species: *Bos taurus*, *Canis familiaris*, *Homo sapiens*, *Monodelphis domestica*, *Macaca mulatta*, *Mus musculus*, *Pan troglodytes*, and *Rattus norvegicus*. The negative set, on the other hand, was assembled collecting the tRNAs and the annotated coding sequences of *Sus scrofa*. The trained SVM was then applied to all candidates.

Finally, we selected only those candidates that presented a putative binding site for the Drosha protein. To perform this search, we used Interagon miRNA SVM software [47].

#### RAKE Peak Identification

The RAKE experimental setup associates fluorescence levels to 16 alternative starts and 22 alternative ends of each predicted miRNA that derives from the set of tailed probes designed and synthesized in the custom microarrays for each predicted pre-miRNA. To identify the most probable endpoints, we then need to analyze these values, looking for spots with a fluorescence level significantly higher than the rest of the group. We implemented this search with a bootstrap approach. Given a group of 22 measures, we extracted one of them in turn and built 10,000 simulated groups by sampling the remaining 21 values with replacement. The number of samples with an average fluorescence level lower than the value of the observed group, divided by 10,000, was taken as an estimate of the probability of having picked a true endpoint for the miRNA. For the rest of the analysis, we retained only the results with a probability higher than 0.75 for the 3′-end and 0.65 for the 5′-end. For a minor group of miRNAs, the probability threshold for the 5′-end was kept higher than 0.58. 3′-ends of miRNAs were confirmed by 4 independent experiments, while the 5′-ends were confirmed by 3 independent experiments.

### Microarrays synthesis, RAKE experiments, qRT-PCR, mRNA experiments, data and RNA-seq analysis

MicroRNA microarrays synthesis, RAKE, bioinformatic identification of mRNA genes, mRNA microarray gene expression experiments, and data analysis are described in detail in the Protocol S1. Briefly, mRNAs were identified using Ensembl transcript (Ver. 56) and UniGene (Ver. 38) databases, evaluating the sequence similarity between the two databases. This process allowed the exclusion of UniGene sequences that were already represented in the Ensembl database and that were used for probe design. MicroRNA and mRNA microarrays were synthesized using the Combimatrix platform (Combimatrix). RAKE experiments were based on the Klenow extension of miRNAs perfectly hybridized to array probes that acted as reaction primers.

qRT-PCR was performed in three replicates according to NCode miRNA protocol (Invitrogen) using as reference gene the Met tRNA and the following PCR cycle: 95°C 10 min.; 95°C 15 sec., 54°C 15 sec, 72°C 40 sec for 45 cycles using 7500 Real Time PCR System (Applied Biosystems). Primers used in the PCR amplification are listed in the Table 1. qRT-PCR data were analyzed according to ΔC_t_ method expressing the original gene expression level as 2^−(Ct gene of interest – Ct reference gene)^. Expression levels were referred to the average expression of the analyzed tissues (Figure 3).

**Table 1:** Primers used in the qRT-PCR experiments.

Name	miRNA sequence Specific primer	Primer for cDNA synthesis (45 nt+2 nt; N) sequence 3′ 5′ direction
prediction_10_32424164_32424229_+_3p	CTAGCCTGGGAACCTCCATATGC	NN(T)20CATGAGACGCAACTATGGTGACGAA
prediction_15_14390446_14390503_-_3p	ATTCGACCCCTAGCCTGGGAACC	NN(T)20CATGAGACGCAACTATGGTGACGAA
prediction_16_68474541_68474601_+_5p	GTACACTCCCGGGCAGCC	NN(T)20CATGAGACGCAACTATGGTGACGAA
prediction_2_12972059_12972118_-_5p	GGATCCGAGCCGCGTCTGCAACC	NN(T)20CATGAGACGCAACTATGGTGACGAA
prediction_3_65092217_65092277_-_5p	GCATTGCTGTGAGCTGTGGTGT	NN(T)20CATGAGACGCAACTATGGTGACGAA

Name	FW Primer	RW Primer
Met t-RNA	CAGAGTGGCGCAGCGGAAGC	CGATCCATCGACCTCTGGGTTATG

Underlined are highlighted primers used in the PCR amplification for the specific miRNA (black sequence) retrotranscribed from miRNA enriched population using universal retrotranscription primer showed in 3^th^ column.

Messenger RNA microarrays were hybridized with 4.8 µg of amplified mRNA labeled by adding biotinylated nucleotides. Messenger RNA data were normalized by the quantile method, while miRNA with a modified loess normalization [88].

RNA-seq datasets were downloaded from the GEO database. We retrieved reads from 5 different datasets (GSE28169, GSE14584, GSE17885, GSE24443, GSE30334). We trimmed the reads using cutadapt [89]. We aligned the reads against the hairpin sequences that were identified with the computational approach and from which mature products were experimentally detected using bowtie allowing 3 multiple alignments and up to 2 mismatches [90]. For an unbiased analysis, we discarded all the reads that mapped to transcripts other than miRNAs (as mRNAs, tRNAs, lncRNAs). The same workflow was used for sequences produced in-house through three runs of Ion Torrent sequencing. Libraries from adipose, liver, heart, kidney, and skeletal muscle tissues were prepared as follow: flashPAGE miRNA purified from each tissue were polyadenilated and than used as template for the retrotranscription with an anchored oligod(T) presenting in the 5′ region the sequence to link the DNA on the ion beads. For the retrotranscription the SMART technique, as described in Biscontin and colleagues [91], was used. By this way sequencing primer and sequencing tag was added in the 5′ of the miRNA. After the retrotranscription 18 cycles of PCR amplification were performed using ion-sequencing primer and ion-bead linking primer. A gel size selection was performed and purified DNA was used as template in the emulsion PCR according to the Ion Torrent manual. Produced libraries were charged in the sequencing machine.

### MicroRNA-target prediction

MicroRNA mRNA target pairs were computed using TargetScan Ver. 5.1 [92]. MicroRNA seeds were defined as the portion of the mature miRNA included between nucleotide positions 2 and 7. We used the Ensembl annotations (Ver. 56) to identify the UTRs of the corresponding Ensembl transcripts. In the case of UniGene sequences (Ver. 38), we aligned the representative sequence of each cluster to the porcine genome. For every sequence, we selected the most extended alignment built from consecutive high scoring pairs (HSPs; maximum allowed gap 50,000 kb). UTR coordinates were then defined as the genomic region that covered the last 894 nucleotides towards the 3′-end. We obtained multiple alignments of the UTR regions with at least three other species from the Ensembl Compara (10-way multiple alignment, Ver. 56, with the following species: *S. scrofa*, *B. taurus*, *C. familiaris*, *E. caballus*, *H. sapiens*, *M. mulatta*, *M. musculus*, *P. pygmaeus*, *P. troglodytes*, and *R. norvegicus*). MicroRNA mRNA target predictions were finally filtered, excluding those interactions that presented a target site too close to the UTR-end as suggested by Lewis [92]. MicroRNA targets that presented inverted correlation with miRNAs gene expression [93] were used in the reconstruction of the networks discussed here.

### Network construction

Networks were based on the Biogrid database [74] for the mRNA-mRNA interactions and on mRNA-miRNA inferred interactions. We used human-based official gene symbols to draw networks in the Cytoscape ambient [94] (Protocol S1).

## Supporting Information

Table S1 **Correlation between phylogenetic distance and the presence of homologous miRNAs in the pig genome.**
(XLS)

Table S2 **Results of triplet-SVM analysis and Microprocessor SVM algorithm.**
(XLS)

Table S3 **Responding probes in the experiments for the identification of the 5′-end of miRNAs.**
(XLS)

Table S4 **Expanded information about 1,459 miRNA sequences identified in the pig tissues by RAKE.**
(XLS)

Table S5 **Over imposition of results obtained by Chen et al. and Xie et al. with those obtained in Martini et al.**
(XLS)

Table S6 **Description of miRNA families prevalently expressed in WBC identified according the TAM tool.**
(XLS)

Table S7 **Interaction network.**
(XLS)

Table S8 **Correlation between lincRNA expression and miRNA expression.**
(XLS)

Table S9 **Functional analysis of miRNA targets in skeletal muscle and heart tissues.**
(XLS)

Protocol S1 **Extended information on protocols and results.**
(DOCX)

## References

[1] Wu CI, Li WH (1985) Evidence for higher rates of nucleotide substitution in rodents than in man. Proc Natl Acad Sci U S A 82: 1741–1745. 3856856 10.1073/pnas.82.6.1741 PMC397348

[2] Huang PL (2009) eNOS, metabolic syndrome and cardiovascular disease. Trends Endocrinol Metab 20: 295–302. 19647446 10.1016/j.tem.2009.03.005 PMC2731551

[3] Granada JF, Kaluza GL, Wilensky RL, Biedermann BC, Schwartz RS, et al (2009) Porcine models of coronary atherosclerosis and vulnerable plaque for imaging and interventional research. EuroIntervention 5: 140–148. 19577996 10.4244/eijv5i1a22

[4] Zhang Q, Widmer G, Tzipori S (2013) A pig model of the human gastrointestinal tract. Gut Microbes 4: 193–200. 23549377 10.4161/gmic.23867 PMC3669164

[5] Kragh PM, Nielsen AL, Li J, Du Y, Lin L, et al (2009) Hemizygous minipigs produced by random gene insertion and handmade cloning express the Alzheimer's disease-causing dominant mutation APPsw. Transgenic Res 18: 545–558. 19184503 10.1007/s11248-009-9245-4

[6] Ross JW, Fernandez de Castro JP, Zhao J, Samuel M, Walters E, et al (2012) Generation of an inbred miniature pig model of retinitis pigmentosa. Invest Ophthalmol Vis Sci 53: 501–507. 22247487 10.1167/iovs.11-8784 PMC3292381

[7] Maxmen A (2012) Model pigs face messy path. Nature 486: 453. 22739291 10.1038/486453a

[8] Sandrin MS, Loveland BE, McKenzie IF (2001) Genetic engineering for xenotransplantation. J Card Surg 16: 448–457. 11925025 10.1111/j.1540-8191.2001.tb00549.x

[9] Ekser B, Rigotti P, Gridelli B, Cooper DK (2009) Xenotransplantation of solid organs in the pig-to-primate model. Transpl Immunol 21: 87–92. 18955143 10.1016/j.trim.2008.10.005

[10] Valdes-Gonzalez RA, Dorantes LM, Garibay GN, Bracho-Blanchet E, Mendez AJ, et al (2005) Xenotransplantation of porcine neonatal islets of Langerhans and Sertoli cells: a 4-year study. Eur J Endocrinol 153: 419–427. 16131605 10.1530/eje.1.01982

[11] Kadner A, Chen RH, Adams DH (2000) Heterotopic heart transplantation: experimental development and clinical experience. Eur J Cardiothorac Surg 17: 474–481. 10773573 10.1016/s1010-7940(00)00362-6

[12] Fosse J, Seegers H, Magras C (2009) Prevalence and risk factors for bacterial food-borne zoonotic hazards in slaughter pigs: a review. Zoonoses Public Health 56: 429–454. 19175574 10.1111/j.1863-2378.2008.01185.x PMC7165994

[13] Wellcome Trust Sanger Institute website. Available: http://www.sanger.ac.uk/resources/downloads/othervertebrates/pig.html. Accessed 2013 Jun 15.

[14] Li M, Wu H, Luo Z, Xia Y, Guan J, et al (2012) An atlas of DNA methylomes in porcine adipose and muscle tissues. Nat Commun 3: 850. 22617290 10.1038/ncomms1854 PMC3508711

[15] Dawson HD, Loveland JE, Pascal G, Gilbert JG, Uenishi H, et al (2013) Structural and functional annotation of the porcine immunome. BMC Genomics 14: 332. 23676093 10.1186/1471-2164-14-332 PMC3658956

[16] Fairbairn L, Kapetanovic R, Beraldi D, Sester DP, Tuggle CK, et al (2013) Comparative Analysis of Monocyte Subsets in the Pig. J Immunol 190: 6389–6396. 23667115 10.4049/jimmunol.1300365

[17] Martins RP, Lorenzi V, Arce C, Lucena C, Carvajal A, et al (2013) Innate and adaptive immune mechanisms are effectively induced in ileal Peyer's patches of Salmonella typhimurium infected pigs. Dev Comp Immunol 41: 100–104. 23644015 10.1016/j.dci.2013.04.020

[18] Hulst M, Smits M, Vastenhouw S, de Wit A, Niewold T, et al (2013) Transcription networks responsible for early regulation of Salmonella-induced inflammation in the jejunum of pigs. J Inflamm (Lond) 10: 18. 23590759 10.1186/1476-9255-10-18 PMC3637394

[19] Adler M, Murani E, Brunner R, Ponsuksili S, Wimmers K (2013) Transcriptomic response of porcine PBMCs to vaccination with tetanus toxoid as a model antigen. PLoS One 8: e58306. 23536793 10.1371/journal.pone.0058306 PMC3607572

[20] Freeman TC, Ivens A, Baillie JK, Beraldi D, Barnett MW, et al (2012) A gene expression atlas of the domestic pig. BMC Biol 10: 90. 23153189 10.1186/1741-7007-10-90 PMC3814290

[21] Kim VN, Han J, Siomi MC (2009) Biogenesis of small RNAs in animals. Nat Rev Mol Cell Biol 10: 126–139. 19165215 10.1038/nrm2632

[22] McDaneld TG, Smith TP, Harhay GP, Wiedmann RT (2012) Next-generation sequencing of the porcine skeletal muscle transcriptome for computational prediction of microRNA gene targets. PLoS One 7: e42039. 22848698 10.1371/journal.pone.0042039 PMC3407067

[23] Zhou B, Liu HL, Shi FX, Wang JY (2010) MicroRNA expression profiles of porcine skeletal muscle. Anim Genet 41: 499–508. 20331612 10.1111/j.1365-2052.2010.02026.x

[24] Liu Y, Li M, Ma J, Zhang J, Zhou C, et al (2013) Identification of differences in microRNA transcriptomes between porcine oxidative and glycolytic skeletal muscles. BMC Mol Biol 14: 7. 23419046 10.1186/1471-2199-14-7 PMC3599761

[25] Siengdee P, Trakooljul N, Murani E, Schwerin M, Wimmers K, et al (2013) Transcriptional profiling and miRNA-dependent regulatory network analysis of longissimus dorsi muscle during prenatal and adult stages in two distinct pig breeds. Anim Genet 44: 398–407. 23506348 10.1111/age.12032

[26] McDaneld TG, Smith TP, Doumit ME, Miles JR, Coutinho LL, et al (2009) MicroRNA transcriptome profiles during swine skeletal muscle development. BMC Genomics 10: 77. 19208255 10.1186/1471-2164-10-77 PMC2646747

[27] Huang TH, Zhu MJ, Li XY, Zhao SH (2008) Discovery of porcine microRNAs and profiling from skeletal muscle tissues during development. PLoS One 3: e3225. 18795099 10.1371/journal.pone.0003225 PMC2528944

[28] Timoneda O, Balcells I, Nunez JI, Egea R, Vera G, et al (2013) miRNA expression profile analysis in kidney of different porcine breeds. PLoS One 8: e55402. 23372853 10.1371/journal.pone.0055402 PMC3555835

[29] Li A, Song T, Wang F, Liu D, Fan Z, et al (2012) MicroRNAome and expression profile of developing tooth germ in miniature pigs. PLoS One 7: e52256. 23272230 10.1371/journal.pone.0052256 PMC3525553

[30] Sharbati S, Friedlander MR, Sharbati J, Hoeke L, Chen W, et al (2010) Deciphering the porcine intestinal microRNA transcriptome. BMC Genomics 11: 275. 20433717 10.1186/1471-2164-11-275 PMC2873480

[31] Podolska A, Kaczkowski B, Kamp Busk P, Sokilde R, Litman T, et al (2011) MicroRNA expression profiling of the porcine developing brain. PLoS One 6: e14494. 21253018 10.1371/journal.pone.0014494 PMC3017054

[32] Zhou Y, Tang X, Song Q, Ji Y, Wang H, et al (2013) Identification and characterization of pig embryo microRNAs by Solexa sequencing. Reprod Domest Anim 48: 112–120. 22646905 10.1111/j.1439-0531.2012.02040.x

[33] Lian C, Sun B, Niu S, Yang R, Liu B, et al (2012) A comparative profile of the microRNA transcriptome in immature and mature porcine testes using Solexa deep sequencing. FEBS J 279: 964–975. 22240065 10.1111/j.1742-4658.2012.08480.x

[34] Li M, Liu Y, Wang T, Guan J, Luo Z, et al (2011) Repertoire of porcine microRNAs in adult ovary and testis by deep sequencing. Int J Biol Sci 7: 1045–1055. 21927574 10.7150/ijbs.7.1045 PMC3174389

[35] Curry E, Safranski TJ, Pratt SL (2011) Differential expression of porcine sperm microRNAs and their association with sperm morphology and motility. Theriogenology 76: 1532–1539. 21872314 10.1016/j.theriogenology.2011.06.025

[36] Luo L, Ye L, Liu G, Shao G, Zheng R, et al (2010) Microarray-based approach identifies differentially expressed microRNAs in porcine sexually immature and mature testes. PLoS One 5: e11744. 20805883 10.1371/journal.pone.0011744 PMC2923610

[37] Li H, Xi Q, Xiong Y, Cheng X, Qi Q, et al (2011) A comprehensive expression profile of microRNAs in porcine pituitary. PLoS One 6: e24883. 21969866 10.1371/journal.pone.0024883 PMC3182167

[38] Li HY, Xi QY, Xiong YY, Liu XL, Cheng X, et al (2012) Identification and comparison of microRNAs from skeletal muscle and adipose tissues from two porcine breeds. Anim Genet 43: 704–713. 22497549 10.1111/j.1365-2052.2012.02332.x

[39] Chen C, Ai H, Ren J, Li W, Li P, et al (2011) A global view of porcine transcriptome in three tissues from a full-sib pair with extreme phenotypes in growth and fat deposition by paired-end RNA sequencing. BMC Genomics 12: 448. 21906321 10.1186/1471-2164-12-448 PMC3188532

[40] Xie SS, Li XY, Liu T, Cao JH, Zhong Q, et al (2011) Discovery of porcine microRNAs in multiple tissues by a Solexa deep sequencing approach. PLoS One 6: e16235. 21283541 10.1371/journal.pone.0016235 PMC3026822

[41] Li M, Xia Y, Gu Y, Zhang K, Lang Q, et al (2010) MicroRNAome of porcine pre- and postnatal development. PLoS One 5: e11541. 20634961 10.1371/journal.pone.0011541 PMC2902522

[42] Martini P, Sales G, Calura E, Brugiolo M, Lanfranchi G, et al (2013) Systems Biology Approach to the Dissection of the Complexity of Regulatory Networks in the S. scrofa Cardiocirculatory System. Int J Mol Sci 14: 23160–23187. 24284405 10.3390/ijms141123160 PMC3856112

[43] Nelson PT, Baldwin DA, Scearce LM, Oberholtzer JC, Tobias JW, et al (2004) Microarray-based, high-throughput gene expression profiling of microRNAs. Nat Methods 1: 155–161. 15782179 10.1038/nmeth717

[44] Berezikov E, van Tetering G, Verheul M, van de Belt J, van Laake L, et al (2006) Many novel mammalian microRNA candidates identified by extensive cloning and RAKE analysis. Genome Res 16: 1289–1298. 16954537 10.1101/gr.5159906 PMC1581438

[45] Alexiou P, Maragkakis M, Papadopoulos GL, Reczko M, Hatzigeorgiou AG (2009) Lost in translation: an assessment and perspective for computational microRNA target identification. Bioinformatics 25: 3049–3055. 19789267 10.1093/bioinformatics/btp565

[46] Xue C, Li F, He T, Liu GP, Li Y, et al (2005) Classification of real and pseudo microRNA precursors using local structure-sequence features and support vector machine. BMC Bioinformatics 6: 310. 16381612 10.1186/1471-2105-6-310 PMC1360673

[47] Helvik SA, Snove O Jr, Saetrom P (2007) Reliable prediction of Drosha processing sites improves microRNA gene prediction. Bioinformatics 23: 142–149. 17105718 10.1093/bioinformatics/btl570

[48] Kim J, Cho IS, Hong JS, Choi YK, Kim H, et al (2008) Identification and characterization of new microRNAs from pig. Mamm Genome 19: 570–580. 18548309 10.1007/s00335-008-9111-3

[49] Kim HJ, Cui XS, Kim EJ, Kim WJ, Kim NH (2006) New porcine microRNA genes found by homology search. Genome 49: 1283–1286. 17213910 10.1139/g06-120

[50] Li M, Xia Y, Gu Y, Zhang K, Lang Q, et al (2010) MicroRNAome of porcine pre- and postnatal development. PLoS One 5: e11541. 20634961 10.1371/journal.pone.0011541 PMC2902522

[51] Nielsen M, Hansen JH, Hedegaard J, Nielsen RO, Panitz F, et al (2010) MicroRNA identity and abundance in porcine skeletal muscles determined by deep sequencing. Anim Genet 41: 159–168. 10.1111/j.1365-2052.2009.01981.x 19917043

[52] Suzuki Y, Yeung AC, Ikeno F (2011) The representative porcine model for human cardiovascular disease. J Biomed Biotechnol 2011: 195483. 21253493 10.1155/2011/195483 PMC3022214

[53] Stangl K, Gunther C, Frank T, Lorenz M, Meiners S, et al (2002) Inhibition of the ubiquitin-proteasome pathway induces differential heat-shock protein response in cardiomyocytes and renders early cardiac protection. Biochem Biophys Res Commun 291: 542–549. 11855822 10.1006/bbrc.2002.6476

[54] Dudley AM, Aach J, Steffen MA, Church GM (2002) Measuring absolute expression with microarrays with a calibrated reference sample and an extended signal intensity range. Proc Natl Acad Sci U S A 99: 7554–7559. 12032321 10.1073/pnas.112683499 PMC124281

[55] Miska EA, Alvarez-Saavedra E, Townsend M, Yoshii A, Sestan N, et al (2004) Microarray analysis of microRNA expression in the developing mammalian brain. Genome Biol 5: R68. 15345052 10.1186/gb-2004-5-9-r68 PMC522875

[56] Liang Y, Ridzon D, Wong L, Chen C (2007) Characterization of microRNA expression profiles in normal human tissues. BMC Genomics 8: 166. 17565689 10.1186/1471-2164-8-166 PMC1904203

[57] Cagnin S, Biscuola M, Patuzzo C, Trabetti E, Pasquali A, et al (2009) Reconstruction and functional analysis of altered molecular pathways in human atherosclerotic arteries. BMC Genomics 10: 13. 19134193 10.1186/1471-2164-10-13 PMC2654039

[58] McGregor CG, Carpentier A, Lila N, Logan JS, Byrne GW (2011) Cardiac xenotransplantation technology provides materials for improved bioprosthetic heart valves. J Thorac Cardiovasc Surg 141: 269–275. 21168032 10.1016/j.jtcvs.2010.08.064

[59] Rajani R, Mukherjee D, Chambers JB (2007) Doppler echocardiography in normally functioning replacement aortic valves: a review of 129 studies. J Heart Valve Dis 16: 519–535. 17944125

[60] Reddy AM, Zheng Y, Jagadeeswaran G, Macmil SL, Graham WB, et al (2009) Cloning, characterization and expression analysis of porcine microRNAs. BMC Genomics 10: 65. 19196471 10.1186/1471-2164-10-65 PMC2644714

[61] Brattelid T, Aarnes EK, Helgeland E, Guvag S, Eichele H, et al (2011) The Normalization Strategy is Critical for the Outcome of miRNA Expression Analyses in the Rat Heart. Physiol Genomics 43: 604–10. 21177382 10.1152/physiolgenomics.00131.2010

[62] He L, Thomson JM, Hemann MT, Hernando-Monge E, Mu D, et al (2005) A microRNA polycistron as a potential human oncogene. Nature 435: 828–833. 15944707 10.1038/nature03552 PMC4599349

[63] Zhao JJ, Lin J, Lwin T, Yang H, Guo J, et al (2010) microRNA expression profile and identification of miR-29 as a prognostic marker and pathogenetic factor by targeting CDK6 in mantle cell lymphoma. Blood 115: 2630–2639. 20086245 10.1182/blood-2009-09-243147 PMC2852365

[64] Foucar K, Reichard K, Czuchlewski D (2010) Bone Marrow Pathology, Third Ed. 2.

[65] Kronick MN (2004) Creation of the whole human genome microarray. Expert Rev Proteomics 1: 19–28. 15966795 10.1586/14789450.1.1.19

[66] Sales G, Coppe A, Bisognin A, Biasiolo M, Bortoluzzi S, et al (2010) MAGIA, a web-based tool for miRNA and Genes Integrated Analysis. Nucleic Acids Res 38: W352–359. 20484379 10.1093/nar/gkq423 PMC2896126

[67] Lewis BP, Shih IH, Jones-Rhoades MW, Bartel DP, Burge CB (2003) Prediction of mammalian microRNA targets. Cell 115: 787–798. 14697198 10.1016/s0092-8674(03)01018-3

[68] Ulitsky I, Bartel DP (2013) lincRNAs: genomics, evolution, and mechanisms. Cell 154: 26–46. 23827673 10.1016/j.cell.2013.06.020 PMC3924787

[69] Fatica A, Bozzoni I (2014) Long non-coding RNAs: new players in cell differentiation and development. Nat Rev Genet 15: 7–21. 24296535 10.1038/nrg3606

[70] Xu S, Zhao L, Larsson A, Venge P (2009) The identification of a phospholipase B precursor in human neutrophils. FEBS J 276: 175–186. 19019078 10.1111/j.1742-4658.2008.06771.x

[71] Kikuchi K, Poss KD (2012) Cardiac regenerative capacity and mechanisms. Annu Rev Cell Dev Biol 28: 719–741. 23057748 10.1146/annurev-cellbio-101011-155739 PMC3586268

[72] Yada H, Murata M, Shimoda K, Yuasa S, Kawaguchi H, et al (2007) Dominant negative suppression of Rad leads to QT prolongation and causes ventricular arrhythmias via modulation of L-type Ca2+ channels in the heart. Circ Res 101: 69–77. 17525370 10.1161/CIRCRESAHA.106.146399

[73] Mayr JA, Merkel O, Kohlwein SD, Gebhardt BR, Bohles H, et al (2007) Mitochondrial phosphate-carrier deficiency: a novel disorder of oxidative phosphorylation. Am J Hum Genet 80: 478–484. 17273968 10.1086/511788 PMC1821108

[74] Stark C, Breitkreutz BJ, Chatr-Aryamontri A, Boucher L, Oughtred R, et al (2011) The BioGRID Interaction Database: 2011 update. Nucleic Acids Res 39: D698–704. 21071413 10.1093/nar/gkq1116 PMC3013707

[75] Ambros V, Bartel B, Bartel DP, Burge CB, Carrington JC, et al (2003) A uniform system for microRNA annotation. RNA 9: 277–279. 12592000 10.1261/rna.2183803 PMC1370393

[76] Bissels U, Wild S, Tomiuk S, Holste A, Hafner M, et al (2009) Absolute quantification of microRNAs by using a universal reference. RNA 15: 2375–2384. 19861428 10.1261/rna.1754109 PMC2779673

[77] Ma Y, Chen Y, Yang Y, Chen B, Liu D, et al (2013) Proteasome inhibition attenuates heart failure during the late stages of pressure overload through alterations in collagen expression. Biochem Pharmacol 85: 223–233. 23142711 10.1016/j.bcp.2012.10.025

[78] Miller E, Hoschler K, Hardelid P, Stanford E, Andrews N, et al (2010) Incidence of 2009 pandemic influenza A H1N1 infection in England: a cross-sectional serological study. Lancet 375: 1100–1108. 20096450 10.1016/S0140-6736(09)62126-7

[79] Hayashita Y, Osada H, Tatematsu Y, Yamada H, Yanagisawa K, et al (2005) A polycistronic microRNA cluster, miR-17-92, is overexpressed in human lung cancers and enhances cell proliferation. Cancer Res 65: 9628–9632. 16266980 10.1158/0008-5472.CAN-05-2352

[80] Cox MB, Cairns MJ, Gandhi KS, Carroll AP, Moscovis S, et al (2010) MicroRNAs miR-17 and miR-20a inhibit T cell activation genes and are under-expressed in MS whole blood. PLoS One 5: e12132. 20711463 10.1371/journal.pone.0012132 PMC2920328

[81] McGraw-Hill (2005) McGraw-Hill concise encyclopedia of bioscience. New York: McGraw-Hill. 972 p.

[82] Schwarz DS, Hutvagner G, Du T, Xu Z, Aronin N, et al (2003) Asymmetry in the assembly of the RNAi enzyme complex. Cell 115: 199–208. 14567917 10.1016/s0092-8674(03)00759-1

[83] Guo L, Lu Z (2010) The fate of miRNA* strand through evolutionary analysis: implication for degradation as merely carrier strand or potential regulatory molecule? PLoS One 5: e11387. 20613982 10.1371/journal.pone.0011387 PMC2894941

[84] Pillai RS (2005) MicroRNA function: multiple mechanisms for a tiny RNA? RNA 11: 1753–1761. 16314451 10.1261/rna.2248605 PMC1370863

[85] Lu Y, Zhang Y, Wang N, Pan Z, Gao X, et al (2010) MicroRNA-328 contributes to adverse electrical remodeling in atrial fibrillation. Circulation 122: 2378–2387. 21098446 10.1161/CIRCULATIONAHA.110.958967

[86] Altschul SF, Gish W, Miller W, Myers EW, Lipman DJ (1990) Basic local alignment search tool. J Mol Biol 215: 403–410. 2231712 10.1016/S0022-2836(05)80360-2

[87] Hofacker IL, Priwitzer B, Stadler PF (2004) Prediction of locally stable RNA secondary structures for genome-wide surveys. Bioinformatics 20: 186–190. 14734309 10.1093/bioinformatics/btg388

[88] Risso D, Massa MS, Chiogna M, Romualdi C (2009) A modified LOESS normalization applied to microRNA arrays: a comparative evaluation. Bioinformatics 25: 2685–2691. 19628505 10.1093/bioinformatics/btp443

[89] Martin M (2011) Cutadapt removes adapter sequences from high-throughput sequencing reads. EMBnet J 17: 10–12.

[90] Langmead B, Trapnell C, Pop M, Salzberg SL (2009) Ultrafast and memory-efficient alignment of short DNA sequences to the human genome. Genome Biol 10: R25. 19261174 10.1186/gb-2009-10-3-r25 PMC2690996

[91] Biscontin A, Casara S, Cagnin S, Tombolan L, Rosolen A, et al (2010) New miRNA labeling method for bead-based quantification. BMC Mol Biol 11: 44. 20553585 10.1186/1471-2199-11-44 PMC2900262

[92] Lewis BP, Burge CB, Bartel DP (2005) Conserved seed pairing, often flanked by adenosines, indicates that thousands of human genes are microRNA targets. Cell 120: 15–20. 15652477 10.1016/j.cell.2004.12.035

[93] Sales G, Coppe A, Bisognin A, Biasiolo M, Bortoluzzi S, et al (2010) MAGIA, a web-based tool for miRNA and Genes Integrated Analysis. Nucleic Acids Res 38: W352–359. 20484379 10.1093/nar/gkq423 PMC2896126

[94] Shannon P, Markiel A, Ozier O, Baliga NS, Wang JT, et al (2003) Cytoscape: a software environment for integrated models of biomolecular interaction networks. Genome Res 13: 2498–2504. 14597658 10.1101/gr.1239303 PMC403769

