# Supplementary material for: Tissue-Specific Expression and Regulatory Networks of Pig MicroRNAome
Source: PLoS One. 2014 Apr 3;9(4):e89755. doi: 10.1371/journal.pone.0089755 (PMC3974652; doi:10.1371/journal.pone.0089755)
Supplement: Protocol S1 — Extended information on protocols and results. (DOCX) [file pone.0089755.s010.docx]

**Tissue-specific expression and regulatory networks of pig microRNAome**

Martini P.^1,2^ *, Sales G.^1^ *, Brugiolo M.^1,2^, Gandaglia A.^3^, Naso F.^3^, De Pittà C.^1^, Spina M.^4^, Gerosa G.^3^, Chemello F. ^1^, Romualdi C.^1^, Cagnin S.^1,2^ #, Lanfranchi G.^1,2^ #

^1^ Department of Biology, University of Padova, Padova, Italy.

^2^ CRIBI Biotechnology Centre, University of Padova, Padova, Italy.

^3^ Department of Cardiac, Thoracic and Vascular Sciences, University of Padova, Padova, Italy.

^4^ Department of Biomedical Sciences, University of Padova, Padova, Italy.

* Contributed equally

# Corresponding author: C.S. e-mail: stefano.cagnin@unipd.it and L.G. e-mail: gerolamo.lanfranchi@unipd.it

**RESULTS**

**Messenger RNA (mRNA)**

Messenger RNA microarray probe design: Ensembl transcripts (Ver. 56) and UniGene (Ver. 38) pig sequences were used to produce a dedicated microarray platform for monitoring mRNA expression. On the basis of sequence similarity, UniGene features that overlapped more than 40% with an Ensembl transcript were discarded. After this filter, we obtained 40,267 UniGene clusters and 19,603 Ensembl transcripts (protein coding + pseudogenes + retrotransposed elements). For this selected collection of sequences we designed microarray probes with different specificity and different distances from the transcript 3’-end, using 6 different algorithms. The two best probes for each sequence, as determined by the reliability of the prediction algorithm and their vicinity to the 3’-end, were experimentally tested in a hybridization trial performed with a pool of mRNA populations independently prepared from 20 pig tissues (GEO: GSE28636). For each transcript with a replicated probe, we selected the probe that was the most responsive and specific on the basis of the intensity of fluorescence in the hybridization test, as suggested by Kronick [1]. The resulting pig whole-genome microarray, here used for the gene expression analysis, is composed of i) 17,048 replicated probes and 963 single probes specific for the Ensembl transcripts, ii) 11,363 replicated probes specific for the UniGene clusters of length comprised between 778 nt and 1,348 nt (Figure SM1), and iii) 28,790 single probes specific for the remaining UniGene clusters. We tried to maintain the mapping of probes as proximal to the 3’-end of the target sequences as possible (Figure SM2 A and SM2 B). As a result, 98.2% of the probes of the Ensembl transcripts mapped within 2,000 nt from their 3’-end while the percentage for UniGene clusters reached 99.9%. Our analysis was not able to identify specific probes for 114 UniGene clusters and 1,592 Ensembl transcripts.

3’ untranslated region (UTR): Since miRNA activity is prevalently based on their interaction with the 3’-UTR of the mRNAs we enriched Ensembl UTR definitions with those for UniGene clusters. According to the distribution of the Ensembl 3’-UTR lengths (mean: 336.83 nt; standard deviation: 278.83 nt) we defined as 3’-UTR the 894 nt region upstream the end of the UniGene sequences (mean + 2 * standard deviation).

**Messenger RNA clusters identification**

The microarray experiments identified eight different mRNA clusters presenting a predominant expression in the following groups of pig tissues: a) tongue, atrium and ventricle, skeletal muscle (all with an high percentage of cells with contractile properties), b) white blood cells, lymph node and spleen (with immune properties), c) liver and kidney (with detoxification and homeostatic roles). A gene enrichment approach applied to these clusters demonstrated specificity of the microarray platform and evidenced major molecular mechanisms in the studied tissues (Figure SM3). The “skeletal muscle contraction” is the biological process to which belong the majority of transcripts highly expressed in skeletal muscle while the “Oxydative phospohorylation” results for those expressed in the heart. This is the consequence of a much richer supply of mitochondria in the heart than skeletal muscle tissue that reflects its greater dependence from ATP for cellular respiratory activity. Tissues involved in the inflammation response were enriched for “immune response” biological process, while liver had “Triglyceride metabolic process” and “complement activation” as the most represented biological function. The liver is, in fact, involved in detoxification, protein synthesis, and production of molecules necessary for digestion and glycogen storage. Moreover, the complement system consists of a number of small blood proteins that are generally synthesized by the liver. Kidney specific genes did not allow the identification of particular biological processes probably because of the low number of functionally identified transcripts, but progesterone receptor membrane component (*PGRMC1*), beta-ureidopropionase (*UPB1*) prevalently expressed in the liver and in the kidney [2] and uromodulin (*UMOD*) were the most abundant transcripts in the kidney.

**MicroRNA tissue-specific expression signatures**

*LIVER:* We identified a specific cluster with miRNA prevalently expressed in the liver (Figure SM4). However, many miRNAs highly expressed in the liver are also expressed at high level in adipose tissue indicating their functional relationship. The pig can provide a valuable supply for livers needed for xeno-transplantation in patients with liver failure [3,4]. Considering the central metabolic role of this organ and the number and broad diffusion of human pathologies that affect the liver, it would very important to study the role of liver-specific miRNA using the pig model. Our data show that there is a high similarity between the miRNA signatures of liver and adipose tissue that may be due to common involvement in lipogenesis (Figure SM4). These concordant miRNAs represent therefore important targets in the studies for metabolic diseases of the liver and for obesity. For example, the highly expressed mir-143 (Figure SM4) was already associated with obesity [5].

**METHODS**

**Computational identification of miRNAs**

The bioinformatic approach for the conserved and *de-novo* identification of pre-miRNAs is fully described in the Methods section of the manuscript.

**Microarrays synthesis**

For this study we synthesized four different types of microarrays platforms: a) two 90K Combimatrix microarrays for the identification of the 3’-end of the predicted miRNAs (GEO: GPL13319, GPL13320); b) the 12K Combimatrix microarray for the identification of the 5’-end of the miRNAs whose 3’-end was successfully determined (GEO: GPL13321); c) the 4 X 2K Combimatrix microarrays for miRNA expression profiling in 14 pig tissues (GEO: GPL13322); d) the 90K Combimatrix microarrays (GEO: GPL13259) for mRNA expression profiling in the same 14 pig tissues used in the miRNA expression profiling. All microarrays were synthesized using the Combimatrix oligonucleotide synthesizer station (Combimatrix) that allows *in-situ* synthesis of the oligonucleotide probes through the phosphoramidites chemistry. All synthesized microarray platforms were tested for uniformity of the probes as suggested by the Combimatrix company.

Microarrays for the identification of the 3’-end of the miRNAs are composed of tailed probes designed to cover each arm of the stem–loop structure of the predicted pre-miRNA (22 probes for each arm shifted by one nucleotide). We adopted this strategy because it is difficult to bioinformatically predict which strand of the pre-miRNA will actually code for the mature miRNA. Moreover, this type of tailing design allows the identification of potential miRNA star (miRNA*) (Figure SM5).

The 12K microarray for the identification of the miRNAs 5’-end were produced following the same strategy used to identify the 3’-end, but using 16 tailed probes (Figure 2 A of the manuscript).

The 4 X 2K microarrays contain specific probes for the 3’-end of the miRNAs. Each specific probe is flanked by a background probe used in the analysis to subtract the correspondent background fluorescence signal (Figure SM6 A).

Each microarray was utilized for up to four experiments, taking advantage from the Combimatrix technology that allows the re-use of microarrays after a stripping procedure to remove the hybridized target. The stripping is characterized by three steps: 1) incubation of the microarray at 65° C in the stripping solution (Combimatrix) for 1.5 hours (90K microarrays) and for 1 hour (12K and 4 X 2K microarrays); 2) washing with 99% ethanol; 3) renaturation with PBS 1X at 65° C for 20 min. Stripped microarray was scanned to evaluate the presence of residual fluorescence evidencing no residual fluorescence (Figure SM6 B).

**RNA extraction**

Tissue samples were taken from three different adult pigs (12 months old) and stored in RNAlater (Ambion) until RNA extraction. Total RNA and small RNAs were extracted independently from each tissue sample by TRIzol reagent (Invitrogen) in association with PureLink miRNA isolation kit (Invitrogen) and flashPAGE instrument. Briefly, approximately 200 mg of tissue was homogenized in 3.5 ml of TRIzol using a tissue homogenizer (IKA Werke). After chloroform addition and centrifugation, the colorless upper aqueous RNA containing phase was mixed with 96-100% ethanol to a final concentration of 35% of ethanol and loaded to PureLink membrane (Invitrogen) to separate total and small RNAs following the manufacturer instructions. Total and small RNAs were quantized using the Nanodrop ND 1000 spectrophotometer (Thermo Fisher Scientific). Equal amounts of RNA prepared from the three pigs and derived from the same tissues were pooled together. 1 ug aliquots were used for total RNA pools, whereas 1.5 ug for small RNA pools. Pooled total RNA samples were tested for quality on Agilent Bioanalizer 2100 using RNA 6000 Nano LabChip. Samples have an average RNA Integrity Number (RIN) of 7.22 (standard deviation = 1). Small RNAs were tested with the 2100 Small RNA to verify the quantity of miRNAs in the sample. MicroRNAs percentage in the small RNA population was comprised between 5% of the white blood cells and 40% of the lymph node (median 22%). After testing miRNA were selected through flashPAGE instrument.

**RAKE experiments**

Identification of miRNA 3’-end: Both 90K microarrays were hybridized with a pool of small RNA preparations from 20 different tissues (superior vena cava, adipose tissue, lung, spleen, stomach, liver, intestine, kidney, descending aorta, left atrium, left ventricle, skeletal muscle, pulmonary aorta, skin, tongue, ascending aorta, arterial derived white blood cells, venal derived white blood cells, coronary valve, lymph node). The pool was assembled adding 300 ng aliquots of miRNAs from each “pooled tissue” (pooled tissue indicates that RNA derives from tissue samples of three different pigs; see RNA samples extraction). Each RAKE test was replicated four times to strengthen the consistency of the identified 3’-end of miRNAs (GEO: GSE28137 and GSE28138). Each replica experiment was preceded by a microarray-stripping procedure (see above). Microarrays were treated for 2 probed with 2 hours at 37° C with a pre-hybridization solution containing SSPE 6X and BSA 8 mg/ml and then hybridized with 300 ng of miRNA pool for 20 hours at 37° C in a static hybridization oven (hybridization buffer: SSPE 6X; BSA 8 mg/ml; 300 ng of small RNAs and spike-in; Table SM1). After hybridization microarrays were washed with the following stringent procedure:

- 1 minute at room temperature with 6x SSPET (SSPE added with 0.05% of Tween-20);
- 1 minute at room temperature with 3x SSPET;
- 1 minute at room temperature with PBS 2X;
- 1 minute at room temperature with Buffer 2, 1X (the buffer for the klenow enzyme).

The RAKE reaction was next performed at 36.5° C by incubating the microarray for 1.5 hours in the following solution: Biotin-14-dATP (Invitrogen) 16 μM; Klenow Fragment (3´→5´ exo–) (NEB) 0.25 U/μl in 1X Buffer 2. Microarrays were washed two times with 1X Buffer 2 and incubated with the biotin blocking solution (Tween-20 0.1% and BSA 10 mg/ml in PBS 2X) for 1 hour at room temperature. Extended miRNAs (primers) were labeled by incubating the microarray with the Dye labeling solution (Tween-20 0.1% ; BSA 10 mg/ml and 1.6 ng of Amersham Cy3-streptavidin in PBS 2X) for 1 hour at room temperature. Microarrays were rinsed with PBST (Tween-20 0.1% in PBS 2X) for 1 minute at room temperature and with PBS 2X for 1 minute at room temperature and scanned with the VersArray microarray confocal laser scanner (Biorad) set at 3 μm resolution. Each microarray was subjected to three consecutive scans at low, medium and high photomultiplier settings [6]. This protocol produces twelve 16-bit tif images, grouped in low, intermediate and high intensity scans. Each group was analyzed separately and only peaks confirmed in all four experiments inside the considered group (low, medium or high) were considered as true peaks.

Identification of miRNA 5’-end: The strategy for the identification of the 5’-end of miRNAs was the same used for the identification of the 3’-end; however, the 12K microarray was probed with the retrotranscribed miRNAs in three independent experiments (Figure 2 A of the manuscript) (GEO: GSE28139). A poly(A) tail was added to the same miRNAs pool used for the identification of the 3’-end of miRNAs. 500 ng of small RNAs was added to a 100 μl polyadenilation solution containing 2.5 mM MnCl_2_; 1mM ATP; 0.08 U/μl E-Polyadenylase Polymerase (Invitrogen) in 1X E-PAP Buffer and incubated for 1 hour at 37° C. MiRNAs were then precipitated using Na-Acetate and 4 volumes of ethanol. Tailed miRNAs were retrotranscribed with Superscript III enzyme (Invitrogen) that lacks terminal-transferase activity. Retrotranscription was performed incubating the mix in 50 μl of 1X First strand Buffer containing 1 mM DTT; 0.5 mM dNTPs; 8 U/μl Superscript III enzyme (Invitrogen) for 1 hour at 42° C. After the RAKE, the microarrays were consecutively scanned three times at low, medium and high settings of photomultiplier [6]. We therefore produced with this protocol nine 16-bit tif images, grouped in low, intermediate and high intensity scans. Each group was analyzed separately and only peaks confirmed in all three experiments inside the considered group (low, medium or high) were taken as true peaks.

**Data analysis of RAKE experiments for miRNA termini identification (peaks identification)**

The 16 bit tiff images obtained from the microarray scanning in each RAKE test were quantized using the Combimatrix imager software. Raw quantization files were uploaded at the GEO database (GSE28137, GSE28138 and GSE28139). Median fluorescence intensity of each group of tailed probes designed for a single predicted pre-miRNA was considered to detect and calculate the probe(s) significantly responsive and therefore diagnostic for the actual miRNA termini. The algorithm used to this purpose is described in the method section of the manuscript. The algorithm identifies positive signal (responsive spots) in the tailed probes for the putative pre-miRNA by comparing the average fluorescence intensity of the entire set of probes of the same tailed group with the averaged intensities of the same set obtained excluding one single probe of the group at a time. This calculation allows measuring the influence of a single probe on the average intensity of a tailing group. The probe(s) that contributes to the largest increment of the averaged fluorescence intensities of the set is then identified as the sequence corresponding to the real termini of the miRNA (peaks). This peak detection was considered true only if confirmed in all four replica experiments performed routinely for the miRNA 3’-end identification or in all three replica experiments for the miRNA 5’-end identification. Each group of scans performed at low, medium or high intensity was independently considered. Moreover, we excluded miRNAs with more than three different termini.

**MicroRNA PCR experiments**

To validate the sequences of miRNAs, we performed a PCR amplification using specific primers (Table SM2). We performed the polyadenilation of miRNAs as described in the related paragraph (Identification of miRNA 5’-end) followed by a retrotranscription from a locked oligod(T) primer. cDNA was then used to perform PCR reactions using the protocol described in the following Table.

| **Cycle** | **Temperature [°C]** | **Time** |
| --- | --- | --- |
| Hot start | 95 | 3’00’’  40 cycles |
| Denaturation | 95 | 15’’ |
| Annealing |  | 35’’ |
| Extension | 72 | 20’’ |
| Final extension | 72 | 1’00’’ |
| Reaction block | 4 | 3’ |

The annealing temperature was set to 52°C for specific primers with the Tm between 40°C to 52°C and to 41°C for primers with the Tm between 31°C to 39 °C.

PCR reaction mix was as indicated in the Table below.

| **Component** | **Quantity** | **Final concentration** |
| --- | --- | --- |
| ss-cDNA [~1 μg] | 2.5 μl |  |
| 10x Advantage 2 PCR buffer | 2.5 μl | 1x |
| dNTPs [10mM] | 0.5 μl | 0.2 mM |
| Universal Primer | 0.25 μl | 100 nM |
| miRNA’s Specific Primer | 0.25 μl | 100 nM |
| 50x Advantage Polymerase mix | 0.5 μl | 1x |
| H_2_O nuclease free | 18.5 μl |  |
| Total | 25 μl |  |

We designed miRNA specific primers using the Integrated DNA Technologies internet tool OligoAnalyser 3.1, available at:

http://eu.idtdna.com/analyzer/Applications/OligoAnalyzer/.

Amplified PCR products were evaluated with the 2100 Agilent Bioanalyzer according to the manufacturer protocol for the Agilent DNA 1000 kit. This protocol allows the discrimination of small quantities of double strand DNA (0.1 – 50 ng) 25 nt to 1000 nt long with 5 bp error in the range between 25 – 100 bp (Figure 2 B of the paper) or by agarose gel.

**Data analysis of RAKE experiments for miRNA tissue expression profiling**

RAKE experiments: miRNAs expression in 14 different pig tissues was evaluated using dedicated microarrays composed of specific probes for miRNAs and associated background probes (Figure SM6 A). The choice of the probes for the miRNA expression was based on the results obtained from experiments for the identification of their 3’-end. Our method avoids the use of labeled miRNA target because is based on the elongation by Klenow enzyme of unmodified miRNAs hybridized to perfectly matching probes with biotin dATP. 300 ng of miRNA from each “tissue pool” (RNA from the same tissue from three different pigs) were hybridized to microarray platform to perform RAKE experiments. Experiments were replicated two times (GEO: GSE28140) and quantization of miRNA concentration was based on experiment specific titration curve obtained from the signals of spike RNA introduced in the reaction mixture (Table SM1 and Figure SM7). Before spike-in based miRNA quantization, inter-arrays fluorescence was normalized using cyclic loess method [7] (Figure SM8). This is an algorithm that allows good grade of normalization in miRNA expression experiments, as discussed by Wu [8], exhibiting the best improvement in the reduction of variability and yielding the highest number of significant differentially expressed miRNAs. After inter-arrays normalization the fluorescence intensity of specific probe for the miRNA was subtracted with the corresponding background fluorescence and then used to extrapolate miRNA concentration from the spike-in derived curve. The spike-in curve was extrapolated using the spline interpolation [9]. The spline interpolation is preferred over polynomial interpolation because the interpolation error can be made small even when using low degree polynomials for the spline. Moreover the spline interpolation avoids the problem of Runge's phenomenon, which occurs when interpolating between equidistant points with high degree polynomials.

Hierarchical cluster analysis and identification of tissue-specific miRNAs: only miRNAs that showed an expression value higher than the threshold of 0.51 pM in at least 14 out from 28 experiments were considered for further studies. This filter restricted the analysis to a total number of 864 miRNA containing also 3’ alternative ends. Hierarchical clustering analysis was performed using Pearson distance for both samples and miRNAs cluster identification (Figure 4 of the manuscript) as implemented in the TIGR MultiExperiment Viewer (MeV) [10]. The support of the nodes in the resulting tree was calculated using the jackknife resampling method with 100 iterations. Jackknifing takes each expression vector and excludes randomly a single element. This method produces expression vectors that have one fewer element and this is done to minimize the effect of single outlier values. Cluster-to-cluster distance was calculated with the average distance: the average distance between each member of one cluster to each member of another cluster is used as a measure of cluster-to-cluster distance. The manual integration of the results of the K-Means analysis [11] performed with Pearson correlation and the QT Cluster analysis [12] allowed the identification of miRNAs preferentially expressed in a specific tissue.

**Construction of mRNA microarray platform**

Identification of probes for mRNAs: Ensembl (Ver. 56) and UniGene (Ver. 38) databases were considered to retrieve transcripts expressed in the pig. Ensembl database has 21,567 transcripts (2 Mitochondrial rRNAs, 22 Mitochondrial tRNAs, 557 miRNAs, 143 misc-RNAs, 19,083 protein coding genes, 474 pseudogenes, 116 rRNAs, 46 retrotransposed elements, 732 snRNAs, 392 snoRNAs) while UniGene has 51,576 clusters. We searched for sequence similarity between Ensemble and UniGene cluster consensus to avoid multiple retrieval of the same transcript, we excluded UniGene consensus that shared more than 40% of sequence identity with Ensembl transcripts. We obtained 59,870 sequences (19,603 Ensembl and 40,267 UniGene) that were used as target for the design of complementary probes to synthesize onto a validation Combimatrix microarray (GEO: GPL13411). Since there is no a better software that is able to identify specific probes for all transcripts we used the following six different software in the probe identification precess: YODA [13], Picky [14], ArrayOligoSelector [15,16], OligoPicker [17], OligoFaktory [18] and CommOligo [19]. Two probes per each target sequence were obtained. We verified probes with the BLAST algorithm [20] to measure their specificity for the target mRNA. Probes could be classified in different groups: a) Ensembl-specific, b) not discriminating between Ensembl transcript isoforms, c) UniGene-specific, d) cross hybridizing with different Ensembl transcripts, e) cross hybridizing with different UniGene clusters, f) cross hybridizing with UniGene and Ensembl sequences. To experimentally test them a 90K microarray was synthesized with two probes designed in the most 3’-end of each transcript belonging to the a, b, and c groups (GEO : GPL13411). We preferred, when possible, to use probes obtained with OligoPicker software for a series of reasons. The software selects specific oligonucleotides by skipping regions with contiguous bases common to other sequences. Low-complexity regions are also filtered out to maintain sequence specificity. This program discards oligonucleotides and sequence regions that may form secondary structures, since both the probes and the target sequence should be easily accessible for hybridization and this step allows setting a Tm range for the requested oligonucleotides. To experimentally verify the specificity of the oligo-probes for their mRNA target, the test microarray was hybridized with a pool of amplified mRNAs derived from the same tissues used to detect 3’-end and 5’-end of miRNAs (GEO: GSE28636). Linear amplification, labeling of RNA and hybridization protocols are described in the next paragraph (Messenger RNA (mRNA) microarray experiments and date analysis). Probes that responded strongly to hybridization with test targets have been chosen to synthesize the definitive 90K Combimatrix microarray platform (GEO: GPL13259) used in mRNA gene expression analysis (GEO: GSE27853).

3’-UTR identification: the Untranslated region for each transcript derived from the Ensembl database was extracted from the definition of the same database, while for UniGene derived transcripts we defined the 3’-UTR as the region that contains the last 894 nt of the UniGene sequence. This definition was basied on the average length of 3’-UTR of Ensembl transcripts (336.83 nt and a standard deviation of 278.83 nt). The length of 894 was calculated as: average + 2 * standard deviations.

**Messenger RNA (mRNA) microarray experiments and data analysis**

Tissue expression profiling of mRNAs: Total RNA samples for each pig tissue was obtained by pooling equal quantities of total RNA prepared independently from homogeneous tissue samples collected from three different animals (see paragraph: “RNA extraction”). 1 μg of pooled RNA was linearly amplified and labeled by the addition of biotinilated nucleotides according to the Ambion MessageAmp™ II aRNA Amplification kit (Ambion). The procedure includes reverse transcription with an oligo-dT primer carrying a T7 promoter to produce the first-strand cDNA. After second-strand synthesis and cleanup, the cDNA is used as template for an in vitro transcription reaction to generate high quantity of antisense RNA (aRNA). Biotinilated UTPs were incorporated into the aRNA during the in vitro transcription reaction. Following purification 18 μg of aRNA was fragmented using the Ambion Fragmentetion Kit (Ambion). Intact and fragmented aRNAs were tested on Agilent Bioanalizer 2100 using RNA 6000 Nano LabChip. The size of intact aRNAs raged between 300 and 4,000 nucleotides while fragmented aRNAs raged from 50 and 250 nucleotides (Figure SM9). Fragmented aRNA was hybridized to pre-hybridized 90K Combimatrix microarrays. Pre-hybridization step was performed for 2 hours at 42° C with a solution containing 5X Denhardt’s solution, 100 ng/μl Salmon sperm DNA, 0.05% SDS in 1X Hyb solution prepared as suggested by Combimatrix. Hybridizations were carried out with 4.8 μg of fragmented aRNA, 25% of DI Formammide, 100 ng/μl Salmon sperm DNA, 0.04% SDS in 1X Hybridization solution at 42° C for 18 hours with constant mixing. After hybridization, microarray platforms were washed with:

- 6X SSPET (SSPE added with 0.05% of Tween-20) preheated at 42° C for 5 min.;
- 3X SSPET for 1 min. at room temperature;
- 0.5X SSPET for 1 min. at room temperature;
- PBST for 1 min. at room temperature;

The microarray chamber was than filled with Biotin Blocking solution (see paragraph: “RAKE experiments”) and incubated at room temperature for 1 hour. Labeling was performed by incubating the microarray with the Dye labeling solution (see paragraph: “RAKE experiments”) for 1 hour at room temperature. After the following washing steps:

- PBST for 1 min. at room temperature two times;
- PBS for 1 min. at room temperature.

microarrays were scanned at 3 μm resolution with the VersArray ChiprRader^TM^ (BioRad). Once the scanning was completed, microarrays were stripped as described in the paragraph “Microarray synthesis”**.**

Data analysis of mRNA profiling: Images of hybridized microarray were quantized using the Combimatrix imaging software. Raw data were normalized with the quantile method [21] (Figure SM10). The goal of the quantile method is to normalize the distribution of probe intensities across a set of microarrays. After normalization the fluorescence intensities of probe spots presenting a value lower than the average of median of all negative control probes were set as NA. The negative control probes introduced in the microarrays were used to calculate the background value (filter). Probe spots presenting NA in more than 6 experiments were excluded from data analysis. Before performing the analysis the intensity values of replicated probes were averaged.

Differentially expressed transcripts were identified by a one-way ANOVA analysis performed with 13 different sample groups (Left Atrium, Skin, Liver, White blood cells, Lymph node, Tongue, Spleen, Skeletal muscle, Lung, Kidney, Stomach, Adipose tissue, and Left Ventricle). The threshold value of P was set at 0.01, on the basis of 1,000 permutations. With this setting we identified 3,411 genes differentially expressed at a significant level, while the remaining 27,999 were not significant. Significant genes were used to perform supported tree sample clustering by the jackknife sampling approach.

The identification of genes preferentially expressed in a given tissue was performed by QT cluster analysis [12], considering only the differentially expressed genes. Clusters were calculated using the Pearson correlation.

**Networks construction**

BioGRID interaction repository (Ver. 3.1.72) [22] was used to discuss genetic interactions. Version 3.1.72 of BioGRID includes a curated set of physical and genetic interactions. The total number of deposited non-redundant interactions are 253,138 whereas the raw interactions are 365,574. Only interactions described for human were used to discover networks that were implemented with miRNA-target interactions, considering homologous genes between pig and human organisms. All networks presented are based on the confirmed miRNA sequences by RAKE and sequencing experiments. Moreover identification of miRNA targets were performed basing TargetScan algorithm and anticorrelation expression. MRNA function were performed on the basis of the enrichment score obtained by the analysis of DAVID database [23].

Gene group enrichment score, based on David database [23], is calculated as the geometric mean of all the enrichment P-values (EASE scores) of each annotation term in a given functional group. To emphasize that the geometric mean is a relative score and not an absolute P-value, minus log transformation is applied on the average P-values. A high score for a gene group indicates that members of that group are likely playing important functional roles in a given study. An enrichment score of 1.3 is equivalent to 0.05 P-value in non-log scale [23].

**SM FIGURE LEGENDS**

**Figure SM1.** Distance of probes for mRNA microarray platform from the 3’-end of the UniGene ‘transcripts’. The X axis measures in base pairs (bp) the distance of mRNA probe sequences from the 3’-end (0 position) of UniGene ‘transcripts’. The Y axis indicates the distribution of probes along this distance, measured as probability density. A. Density probability plot for all UniGene clusters. B. Enlargement of the 0 – 4,000 bp region of panel A. The grey area of the curve represents the positions of the replicated probes (11,363) in the microarray. N = total number of targeted UniGene ‘transcripts’.

**Figure SM2.** A. Number of probes for mRNA microarray platform related to the distance from 3’-end of Ensembl transcripts. 98.2% of the probes map within 2,000 nt from 3’-end. B. Number of probes related to the distance from 3’-end of the UniGene clusters. The inserted rectangle shows the enlargement of the region from 0 to 2,000 nt where 99.9 % of the probes are located.

**Figure SM3.** Gene categories that are enriched in tissue-specific clusters of mRNA expression profiles.

**Figure SM4.** Cluster of miRNA presenting the higher expression in the liver. Yellow lateral bars highlight miRNA sub-clusters that sowed a high expression both in the liver and in the adipose tissue supporting their functional relation and their similar miRNA expression.

**Figure SM5.** Probe design description to identify the exact 3’-ends of miRNAs. A. Hairpin of the ENSSSCT00000020014/ssc-mir-136 where the blue rectangle represents the 3p arm and the red one the 5p arm. Black arrows show 0 position respectively in the 3p and 5p arms; green arrows show the ssc-mir-136 termini of the sequence deposited in the miRBase database; yellow arrows show the end positions we find for the miRNA that mature from the described hairpin. Braces below the hairpin indicate sequences complementary to the shifted probes of the -1, 1 and 14 regions respect to the 0 position. B. Fluorescence intensity of each shifted probe. Blue bars show fluorescence of the probes in the 3p arm of the hairpin while red bars the one of 5p arm. All probes complementary to the sequence in the 3p hairpin arm have the same fluorescence intensity while those complementary to the 5p hairpin arm have a peak in the 13 – 16 region with the probe 14 corresponding to the 3’-end of the miRNA that presents the highest fluorescence intensity. C. Sequences of the shifted probes complementary to the sequence of the 5p hairpin’s arm. Sequences are characterized by a common spacer from the microarray surface, the specific sequence (orange) and a d(T) stretch useful for the RAKE experiments (detector). Only miRNA perfectly matched to the specific sequence will be extended by Klenow polymerase in the presence of only biotinilated-dATP.

**Figure SM6.** An explicative portion of the scan of a 4 X 2k Combimatrix microarray after the RAKE and labeling reactions (A) and the stripping step (B). A. Spike-in spot are indicated by red line while blue arrow indicates a specific probe. The orange arrow indicates the background probe correspondent to the specific probe indicated by the blue arrow. Each background probe was positioned on the right of the specific probe.

**Figure SM7.** Example of a titration curve based on the spike-in controls. Spikes are represented by points while the line represents the curve interpolation according to the spline algorithm.

**Figure SM8.** Box plot of miRNA expression profiles obtained with custom microarray platforms. A. Raw expression profiles. B. Normalized expression profiles. Y-axes are in log scale.

**Figure SM9.** Electropherograms of intact and fragmented aRNA. Fragmented aRNA (blue line) are between ~ 50 and ~ 250 nucleotides (nt) while aRNA (red line) between ~ 200 and ~ 3,500 nt. FU: fluorescence units.

**Figure SM10.** Box plot of mRNA expression profiles obtained with custom microarray platforms. A. Raw expression profiles. B. Normalized expression profiles. Y-axes are in log scale.

**SM TABLES**

**Table SM 1. Concentration of spike-in targets in miRNA RAKE experiments**

| **Name** | **Concentration** |
| --- | --- |
| SP_1 | 0,518 pM |
| SP_2 | 0,889 pM |
| SP_3 | 3,7 pM |
| SP_4 | 8,89 pM |
| SP_5 | 18,5 pM |
| SP_6 | 37 pM |
| SP_7 | 51.85 pM |
| SP_8 | 74,1 pM |
| SP_9 | 222 pM |
| SP_10 | 474 pM |
| SP_11 | 889 pM |

Concentrations are expressed in pico moles (pM).

**Table SM2. PCR primers for the miRNAs**

| **Name** | **Sequence**  **miRNA**  **Specific primer** | **Primer for cDNA synthesis (26 nt + 2 nt; N) sequence 3’ 5’ direction** | **PCR amplicon length (bp)** | **Agilent size (bp)** | **Primer name** |
| --- | --- | --- | --- | --- | --- |
| prediction_1_165028452_165028513_-_3p | CTTATCCTTTAGTTAAGAGGAGGAG  TTATCCTTTAGTTAAGAGGAGGAGC  TTAGTTAAGAGGAGGAG | NNTTTTTTTTTTTTTTTGTGCCTGTGAC | 44 | 58 | A |
| prediction_2_51459801_51459868_+_3p | GTTGTTCTAAATTTTTCTTTTCTTTTCTT  GTTGTTCTAAATTTTTCTTTTCT | NNTTTTTTTTTTTTTTTGTGCCTGTGAC | 55 | 60 | B |
| ENSSSCT00000020817_3_29678100_29678195_-_5p | CACCTGGGGATCTTGCACCAAA  CCTGGGGATCTTGCACCAAA  TGGGGATCTTGCAC | NNTTTTTTTTTTTTTTTGTGCCTGTGAC | 44 | 47 | C |
| prediction_4_131579571_131579634_-_3p | GATACCTGGTTGTTAGTGGTGCC  GATACCTGGTTGTTAGTG | NNTTTTTTTTTTTTTTTGTGCCTGTGAC | 49 | 55 | D |
| prediction_8_76593438_76593497_+_3p | GTTAGAAACATACCTGTCAGGTGGGAAGG  GTTAGAAACATACCTGTCA | NNTTTTTTTTTTTTTTTGTGCCTGTGAC | 55 | 57 | E |
| prediction_9_25400874_25400934_-_5p | GTGTATATGTGGCTGCCTTGTACAGGGGG  GTGTATATGTGGCTGC | NNTTTTTTTTTTTTTTTGTGCCTGTGAC | 55 | 53 | F |
| hsa-mir-302c_8_92257532_92257600_+_5p;  ENSSSCT00000021153_8_92257532_92257600_+_5p | GATCCCCTTTGCTTTAACATGGGGGTACC  GATCCCCTTTGCTTTAAC | NNTTTTTTTTTTTTTTTGTGCCTGTGAC | 55 | 50 | G |
| ENSSSCT00000020476_18_5054286_5054404_-_5p | GCCAGGAAGAGGAGGAAGCC  CAGGAAGAGGAGGAA | NNTTTTTTTTTTTTTTTGTGCCTGTGAC | 44 | 44 | H |
| prediction_6_76724242_76724304_+_5p | GGAGCCTGGGATGCC  GGAGCCTGGGATGC | NNTTTTTTTTTTTTTTTGTGCCTGTGAC | 41 | 45 | I |

Continue next page

| **Name** | **Sequence**  **miRNA**  **Specific primer** | **Primer for cDNA synthesis (26 nt + 2 nt; N) sequence 3’ 5’ direction** | **PCR amplicon length (bp)** | **Agilent size (bp)** | **Primer name** |
| --- | --- | --- | --- | --- | --- |
| prediction_11_15371078_15371148_+_5p | ACTGCGCCATGATGGGAACTCCCAGAGAACC  ACTGCGCCATGATG | NNTTTTTTTTTTTTTTTGTGCCTGTGAC | 57 | 55 | L |
| hsa-mir-671_18_5054286_5054404_-_3p | GGCTCCACCTTTTCCGGGCCGTGGAGCCA  CTCCACCTTTTCCGG | NNTTTTTTTTTTTTTTTGTGCCTGTGAC | 53 | 48 | M |
| prediction_16_34897772_34897832_-_3p | TGGAGAGGTGTGGGGAAGCCA  TGGAGAGGTGTGGG | NNTTTTTTTTTTTTTTTGTGCCTGTGAC | 47 | 51 | N |
| prediction_2_76634212_76634269_-_5p | CAGATCCTTTGCCTTTCTGGGACTCGCCA  CAGATCCTTTGCCTTTC | NNTTTTTTTTTTTTTTTGTGCCTGTGAC | 55 | 52 | O |
| prediction_1_274143573_274143630_-_3p | AGCTTTCGGGTCGCC  CTTTCGGGTCGCC | NNTTTTTTTTTTTTTTTGTGCCTGTGAC | 39 | 42 | P |
| prediction_2_20713887_20713938_-_5p | CCATCCGAGGTGCCA  CATCCGAGGTGCCA | NNTTTTTTTTTTTTTTTGTGCCTGTGAC | 40 | 44 | Q |
| prediction_8_29373598_29373658_-_3p | CTCTTAACCTCTTCAACAGGGAGG  CTCTTAACCTCTTCAACAG | NNTTTTTTTTTTTTTTTGTGCCTGTGAC | 50 | 53 | R |
| PCR primers for amplification of miRNAs evaluated by agarose gel | | | | | |
|  |  | **Primer for cDNA synthesis (45 nt + 2 nt; N) sequence 3’ 5’ direction** |  |  |  |
| ggo-mir-23a_2_56861158_56861231_-_3p | AATCACATTGCCAGGGATTTCCAA  AATCACATTGCCAGGGATTTCCAA | NN(T)20CATGAGACGCAACTATGGTGACGAA | 69 |  | ggo-mir-23a |
| prediction_10_32424164_32424229_+_3p | CTAGCCTGGGAACCTCCATATGC  TAGCCTGGGAACCTCCATATGC | NN(T)20CATGAGACGCAACTATGGTGACGAA | 67 |  | P_10_32424164 |
| prediction_15_14390446_14390503_-_3p | ATTCGACCCCTAGCCTGGGAACC  CGACCCCTAGCCTGGGAA | NN(T)20CATGAGACGCAACTATGGTGACGAA | 65 |  | P_15_14390446 |
| prediction_16_68474541_68474601_+_5p | GTACACTCCCGGGCAGCC  GTACACTCCCGGGCAGCC | NN(T)20CATGAGACGCAACTATGGTGACGAA | 63 |  | P_16_68474541 |
| prediction_18_53313483_53313543_+_5p | CAGCTGCCGGCCTACACCACAGCC  CAGCTGCCGGCCTACACC | NN(T)20CATGAGACGCAACTATGGTGACGAA | 69 |  | P_18_53313483 |
| prediction_1_191689912_191689974_+_3p | AGCTCCGATTCGACCCCTAGCCT  AGTTCCGATTCGACCCCTAG | NN(T)20CATGAGACGCAACTATGGTGACGAA | 68 |  | P_1_191689912 |
| prediction_2_12972059_12972118_-_5p | GGATCCGAGCCGCGTCTGCAACC  GGATCCGAGCCGCGTCTGCAA | NN(T)20CATGAGACGCAACTATGGTGACGAA | 68 |  | P_2_12972059 |
| prediction_3_65092217_65092277_-_5p | GCATTGCTGTGAGCTGTGGTGT  GCATTGCTGTGAGCTGTGGTG | NN(T)20CATGAGACGCAACTATGGTGACGAA | 67 |  | P_3_65092217 |
| prediction_5_17349754_17349814_-_5p | CTGTGTCTGTGACCTACACCACAGC  CTGTGTCTGTGACCTACACCACA | NN(T)20CATGAGACGCAACTATGGTGACGAA | 70 |  | P_5_17349754 |
| ptr-mir-25a_6_39533847_39533932_+_3p | TGAGGTTCTTGGGAGCC  GGGTGAGGTTCTTGGGAGC | NN(T)20CATGAGACGCAACTATGGTGACGAA | 65 |  | ptr-mir-25a_6 |
| prediction_1_146333652_146333723_-_5p | CTGTGCCTGTGGCGTAGGCCAGCAGCT  CCTGTGGCGTAGGCCAGC | NN(T)20CATGAGACGCAACTATGGTGACGAA | 67 |  | P_1_146333652 |
| prediction_14_62081641_62081708_-_3p | GTGCTGGGGGAGTACC  GTGCTGGGGGAGTACC | NN(T)20CATGAGACGCAACTATGGTGACGAA | 61 |  | P_14_62081641 |
| prediction_14_61690864_61690920_+_3p | CAGGTTTGATCCCTGGCCT  CAGGTTTGATCCCTGGCCT | NN(T)20CATGAGACGCAACTATGGTGACGAA | 64 |  | B - C |
| ssc-mir-24a_10_26148231_26148306 | TGGCTCAGTTCAGCAGGAACAG  TGGCTCAGTTCAGCAGGAAC | NN(T)20CATGAGACGCAACTATGGTGACGAA | 67 |  | A - D |

Name column contains miRNA name_chromosome_start-pre-miRNA_stop-pre-miRNA_genomic-strand_arm of the hairpin; Sequence column has in black the sequence of the miRNA and in red the sequence of the specific primer for the PCR reaction; Primer for cDNA synthesis contain the sequence of the primer used in the retrotranscription reaction; PCR amplicon length describe the expected length of the PCR amplicon; Agilent size column describe the size of detected PCR amplicon. Agilent chips have an error of 5 bp in the range of 25 – 100 bp. Primer name correspond to the names in the Figure 2 B. In yellow are evidenced amplicons that do not satisfy dimension constraint. About PCR tested on agarose gel, only amplification of prediction_14_61690864_61690920_+_3p and ssc-mir-24a_10_26148231_26148306 was performed on the presence of mRNA producing a smear. This result shows problems in the PCR amplification without performing specific miRNA retrotranscription or miRNA purification. In green are evidenced high confidence confirmed miRNA, in violet those medium confidence and in grey those with low confidence.

**SM FIGURES**

**Figure SM1.**

**
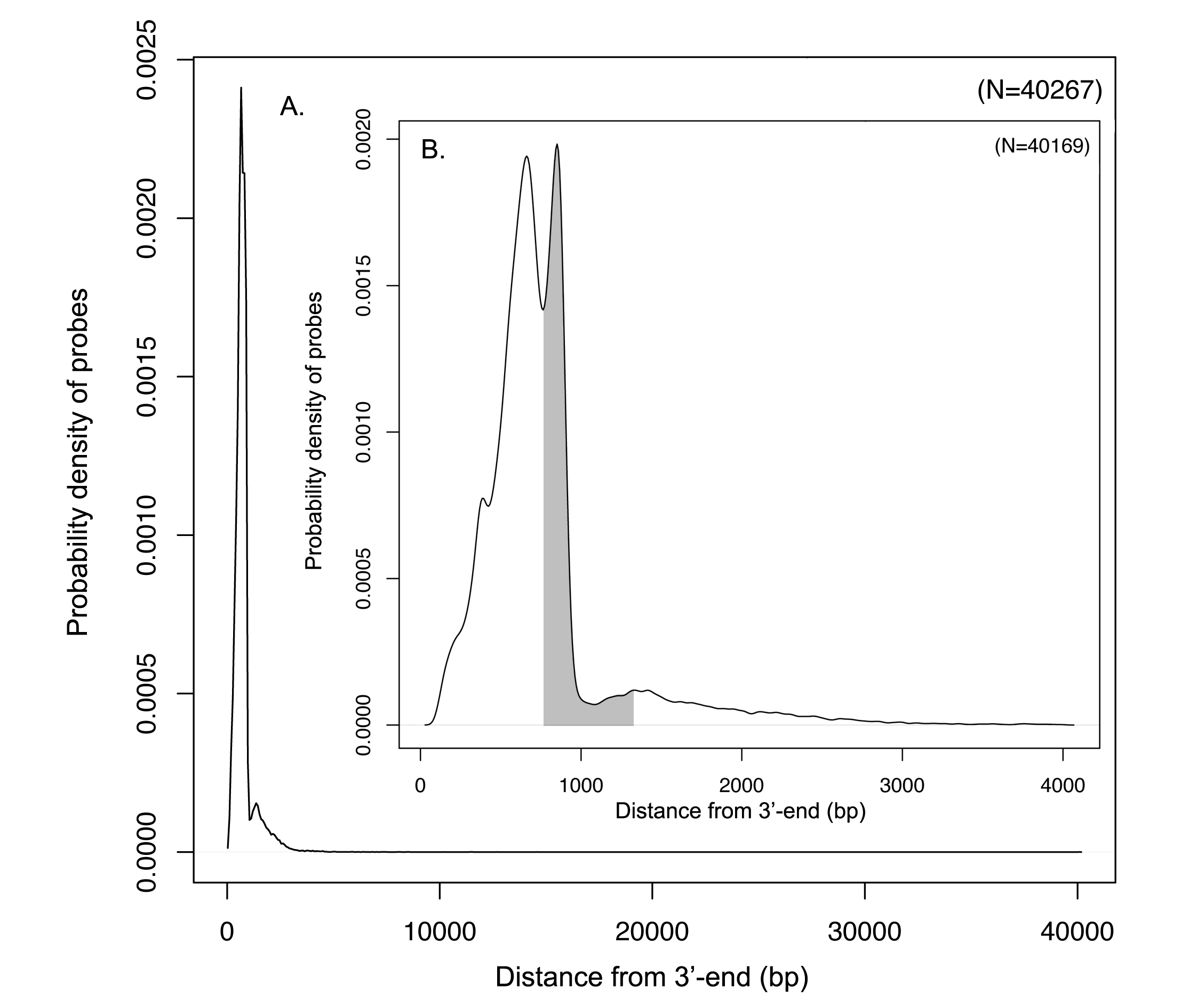
**

**Figure SM2.**

**
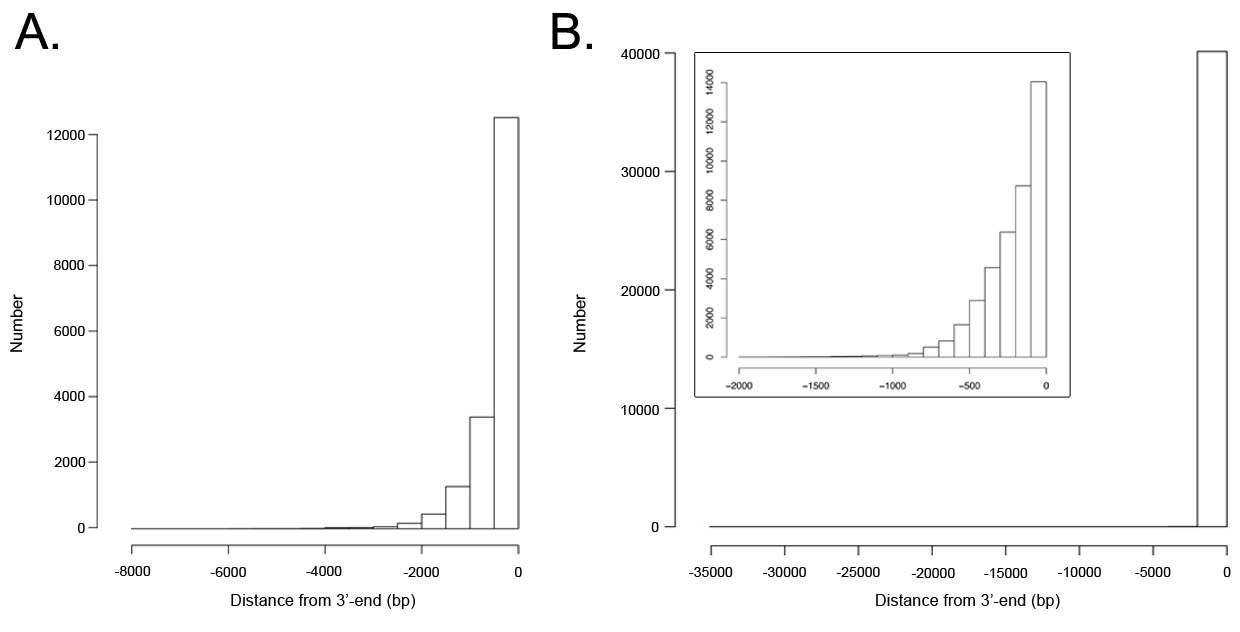
**

**Figure SM3.**

**
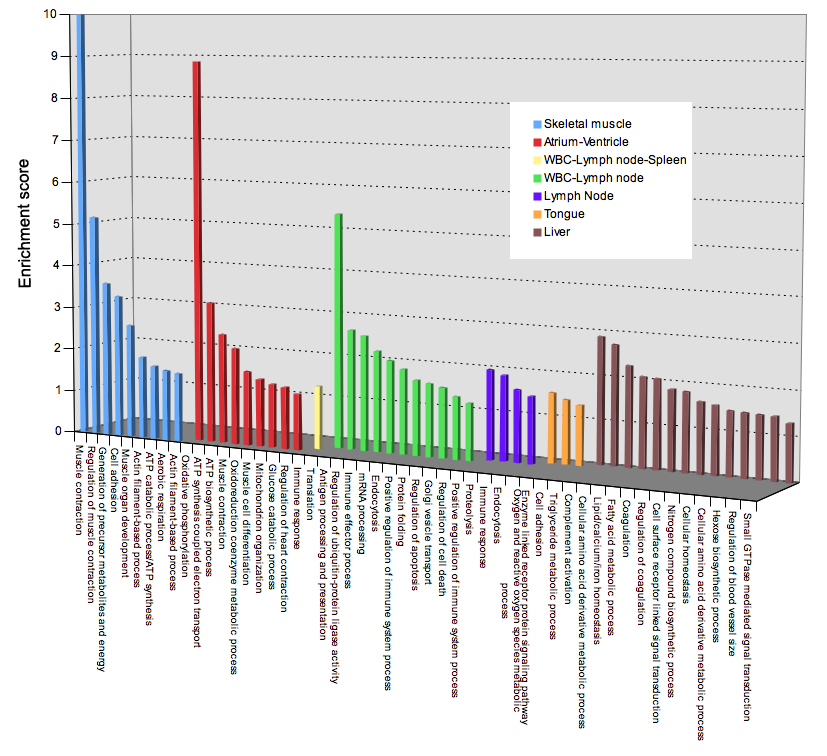
**

**Figure SM4**

**
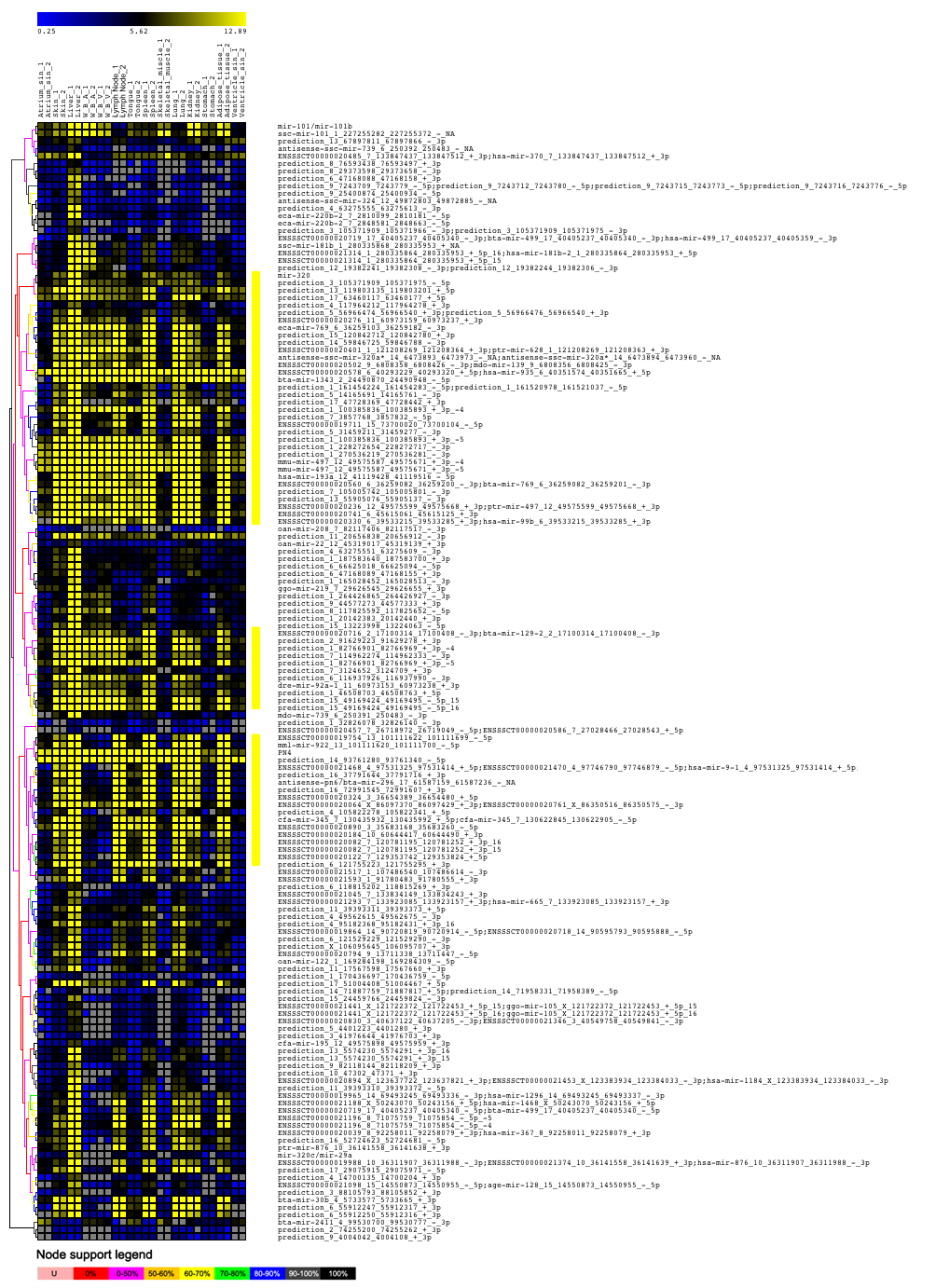
**

**Figure SM5.**

**
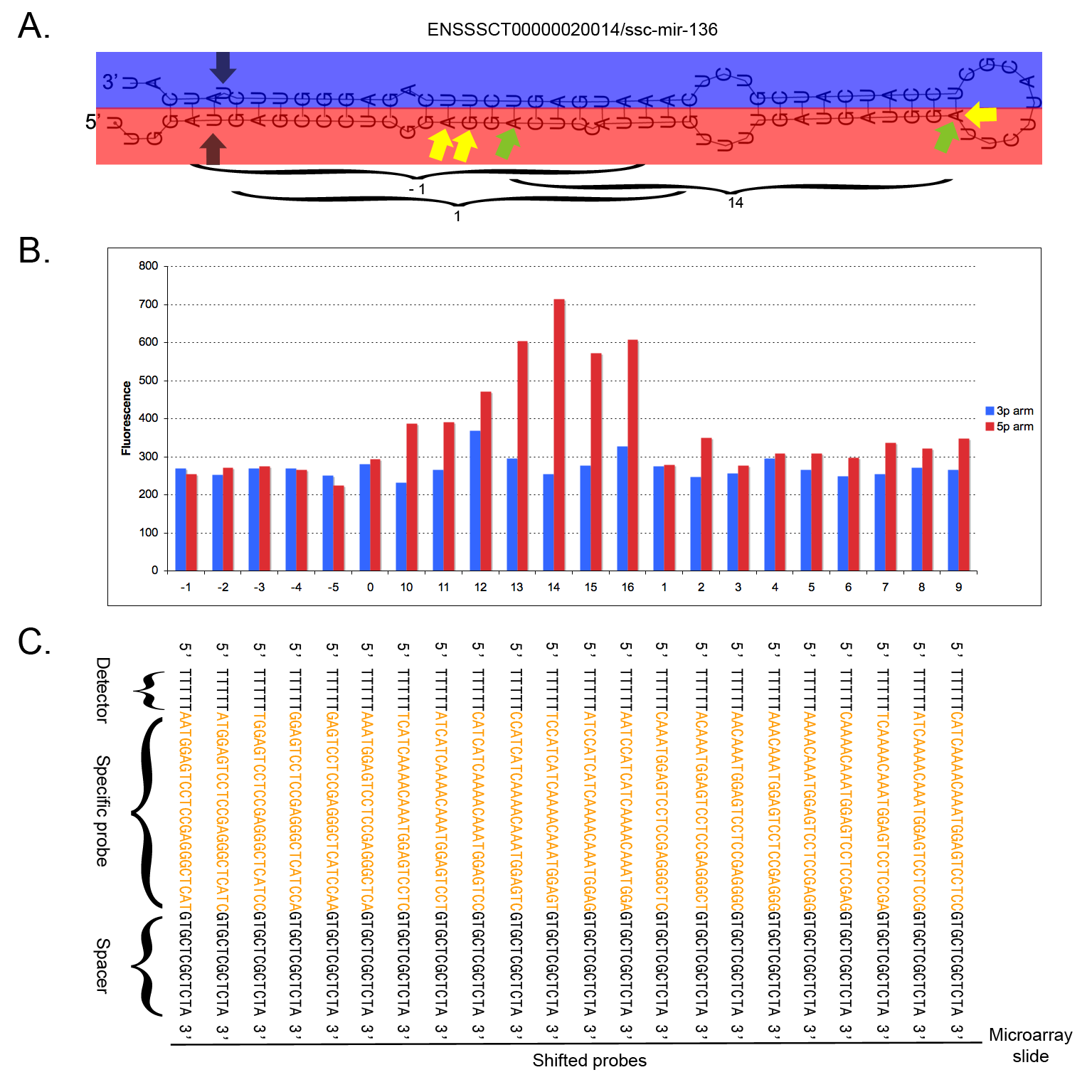
**

**Figure SM6.**

**
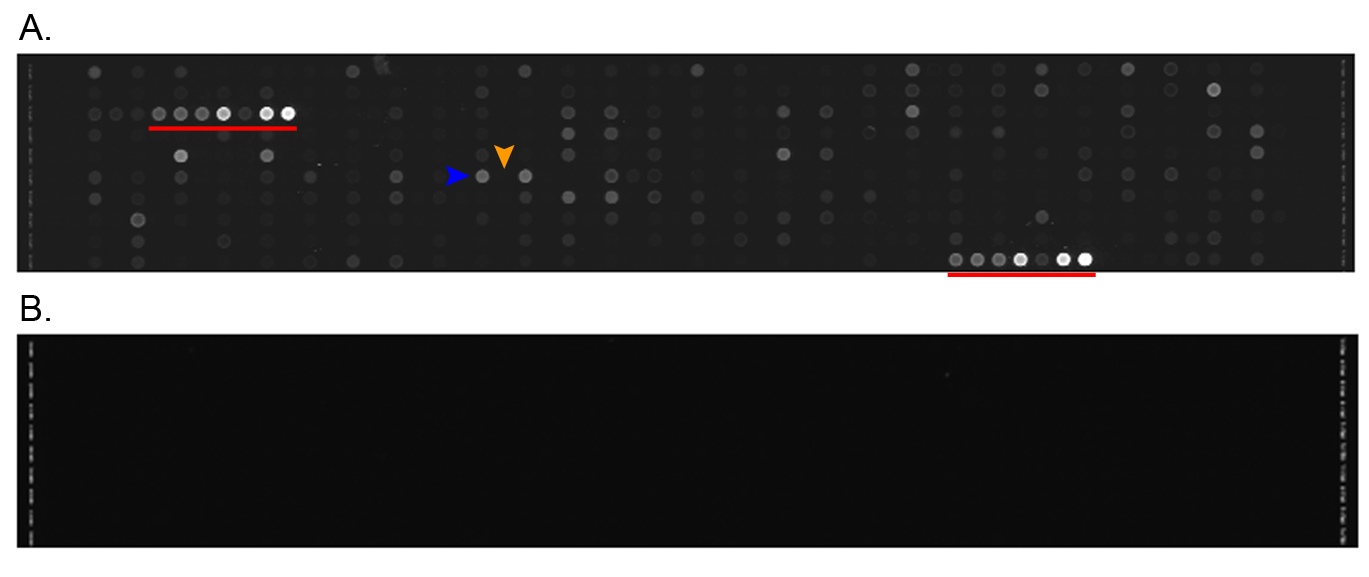
**

**Figure SM7.**

**
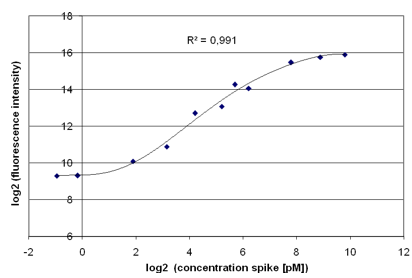
**

**Figure SM8.**

**
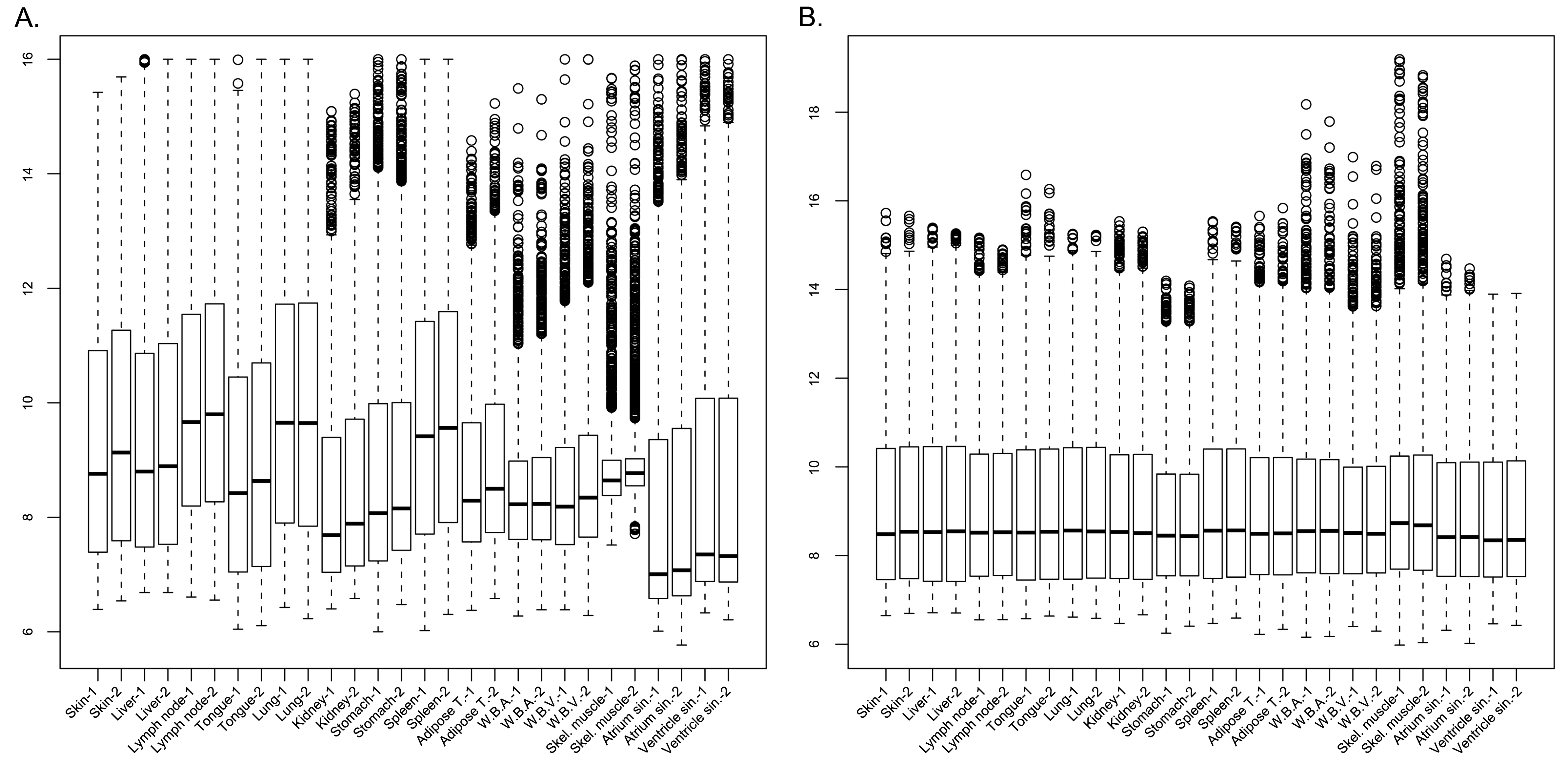
**

**Figure SM9.**

**
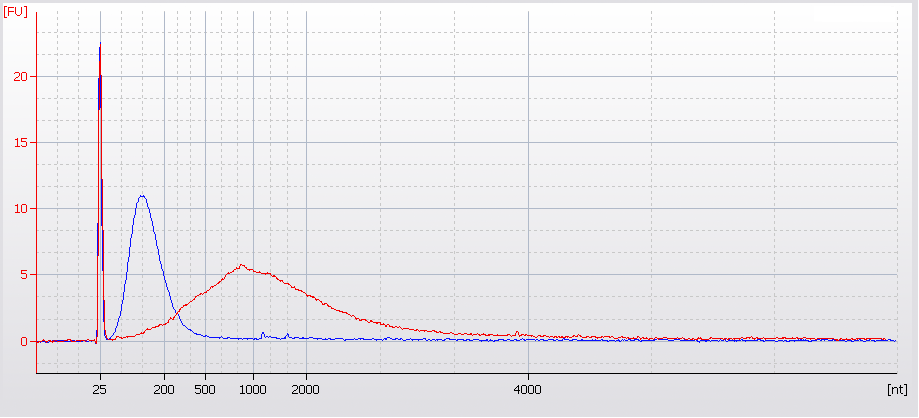
**

**Figure SM10.**

**
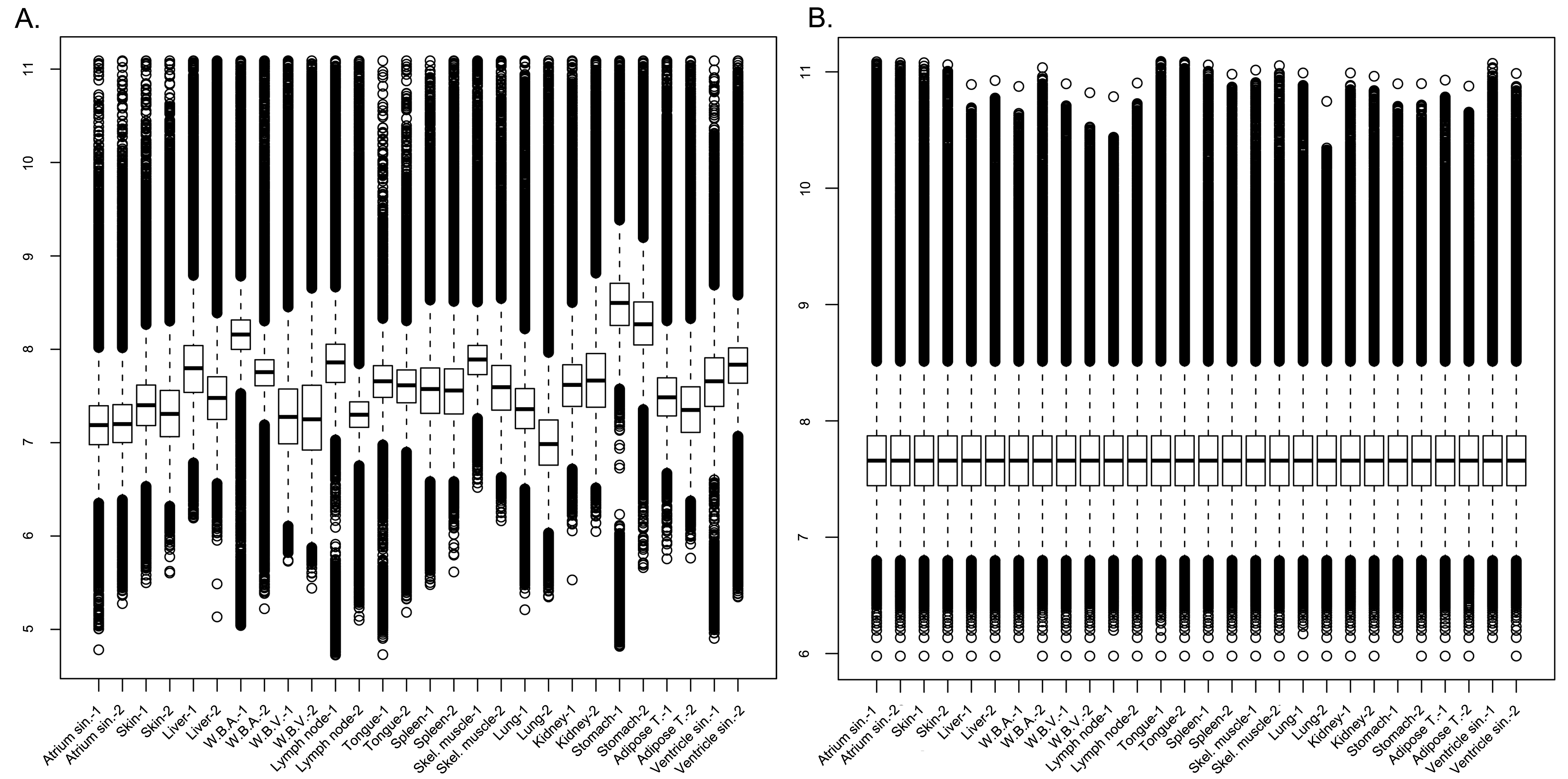
**

**Bibliography**

1. Kronick MN (2004) Creation of the whole human genome microarray. Expert Rev Proteomics 1: 19-28.

2. Sakamoto T, Sakata SF, Matsuda K, Horikawa Y, Tamaki N (2001) Expression and properties of human liver beta-ureidopropionase. J Nutr Sci Vitaminol (Tokyo) 47: 132-138.

3. Ekser B, Gridelli B, Tector AJ, Cooper DK (2009) Pig liver xenotransplantation as a bridge to allotransplantation: which patients might benefit? Transplantation 88: 1041-1049.

4. Hara H, Campanile N, Tai HC, Long C, Ekser B, et al. (2010) An in vitro model of pig liver xenotransplantation--pig complement is associated with reduced lysis of wild-type and genetically modified pig cells. Xenotransplantation 17: 370-378.

5. Takanabe R, Ono K, Abe Y, Takaya T, Horie T, et al. (2008) Up-regulated expression of microRNA-143 in association with obesity in adipose tissue of mice fed high-fat diet. Biochem Biophys Res Commun 376: 728-732.

6. Cagnin S, Biscuola M, Patuzzo C, Trabetti E, Pasquali A, et al. (2009) Reconstruction and functional analysis of altered molecular pathways in human atherosclerotic arteries. BMC Genomics 10: 13.

7. Risso D, Massa MS, Chiogna M, Romualdi C (2009) A modified LOESS normalization applied to microRNA arrays: a comparative evaluation. Bioinformatics 25: 2685-2691.

8. Wu W, Dave N, Tseng GC, Richards T, Xing EP, et al. (2005) Comparison of normalization methods for CodeLink Bioarray data. BMC Bioinformatics 6: 309.

9. Helmuth S (1993) Two Dimensional Spline Interpolation Algorithms. Hardback.

10. Saeed AI, Bhagabati NK, Braisted JC, Liang W, Sharov V, et al. (2006) TM4 microarray software suite. Methods Enzymol 411: 134-193.

11. Soukas A, Cohen P, Socci ND, Friedman JM (2000) Leptin-specific patterns of gene expression in white adipose tissue. Genes Dev 14: 963-980.

12. Heyer LJ, Kruglyak S, Yooseph S (1999) Exploring expression data: identification and analysis of coexpressed genes. Genome Res 9: 1106-1115.

13. Nordberg EK (2005) YODA: selecting signature oligonucleotides. Bioinformatics 21: 1365-1370.

14. Chou HH, Hsia AP, Mooney DL, Schnable PS (2004) Picky: oligo microarray design for large genomes. Bioinformatics 20: 2893-2902.

15. Bozdech Z, Zhu J, Joachimiak MP, Cohen FE, Pulliam B, et al. (2003) Expression profiling of the schizont and trophozoite stages of Plasmodium falciparum with a long-oligonucleotide microarray. Genome Biol 4: R9.

16. Bozdech Z, Llinas M, Pulliam BL, Wong ED, Zhu J, et al. (2003) The transcriptome of the intraerythrocytic developmental cycle of Plasmodium falciparum. PLoS Biol 1: E5.

17. Wang X, Seed B (2003) Selection of oligonucleotide probes for protein coding sequences. Bioinformatics 19: 796-802.

18. Schretter C, Milinkovitch MC (2006) OligoFaktory: a visual tool for interactive oligonucleotide design. Bioinformatics 22: 115-116.

19. Li X, He Z, Zhou J (2005) Selection of optimal oligonucleotide probes for microarrays using multiple criteria, global alignment and parameter estimation. Nucleic Acids Res 33: 6114-6123.

20. Altschul SF, Gish W, Miller W, Myers EW, Lipman DJ (1990) Basic local alignment search tool. J Mol Biol 215: 403-410.

21. Bolstad BM, Irizarry RA, Astrand M, Speed TP (2003) A comparison of normalization methods for high density oligonucleotide array data based on variance and bias. Bioinformatics 19: 185-193.

22. Stark C, Breitkreutz BJ, Chatr-Aryamontri A, Boucher L, Oughtred R, et al. (2011) The BioGRID Interaction Database: 2011 update. Nucleic Acids Res 39: D698-704.

23. Huang da W, Sherman BT, Lempicki RA (2009) Systematic and integrative analysis of large gene lists using DAVID bioinformatics resources. Nat Protoc 4: 44-57.
